# Design, synthesis, structural characterization, and antioxidant potential of novel triazole- and oxadiazole-based hydrazide-hydrazone derivatives: spectroscopic, DFT, and molecular docking studies

**DOI:** 10.1186/s13065-025-01698-6

**Published:** 2025-12-24

**Authors:** Mariam A. Abdo, Safa A. Badawy, Ahmed A. Fadda, Mohamed R. Elmorsy

**Affiliations:** Department of Chemistry, Faculty of Science, Mansoura University, El- Gomhoria Street, Mansoura, 35516 Egypt

**Keywords:** 1,2,4-Triazole derivatives, 1,2,4-Oxadiazole derivatives, Antioxidant activity, Density functional theory (DFT), Molecular electrostatic potential (MEP), Molecular docking

## Abstract

A novel series of hydrazide-hydrazone derivatives incorporating 1,2,4-triazole and 1,2,4-oxadiazole scaffolds was synthesized through multistep condensation and cyclization reactions starting from 4-nitrobenzohydrazide. The structures of the synthesized compounds (**MI-1 to MI-9**) were confirmed by FTIR, ^1^H NMR, and ^13^C NMR spectroscopy, complemented by elemental and mass analyses. Their antioxidant potential was assessed in vitro using the 2,2-diphenyl-1-picrylhydrazyl (DPPH) radical scavenging assay, where several derivatives demonstrated comparable or superior activity to ascorbic acid. To elucidate structure–activity relationships, density functional theory (DFT) calculations were performed to analyze optimized geometries, frontier molecular orbitals (HOMO–LUMO), and molecular electrostatic potential (MEP) surfaces. Furthermore, molecular docking studies against the Keap1–Nrf2 complex revealed favorable binding interactions for select compounds, highlighting their potential as antioxidant modulators through Nrf2 pathway activation. The combined spectroscopic, theoretical, and docking insights provide a comprehensive understanding of the structure–activity relationships of these novel heterocyclic derivatives and support their potential application as antioxidant therapeutic leads.

**Supplementary Information:**

The online version contains supplementary material available at 10.1186/s13065-025-01698-6.

## Introduction

**Fig. 1:**
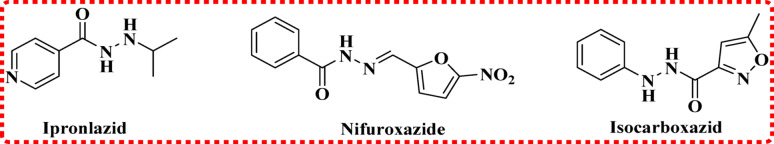
Hydrazide and hydrazone derivatives used as drugs

Hydrazide and hydrazone derivatives represent a versatile class of nitrogen-containing compounds with significant interest in medicinal chemistry. Due to their structural features and ability to participate in diverse biochemical interactions, they exhibit a wide range of pharmacological activities [1]. In benzo-fused N-heterocycles, hydrazone functional groups play a key role in condensation reactions, enabling efficient molecular design strategies [2]. Hydrazide–hydrazone scaffolds have been reported to demonstrate notable antioxidant activity, which is essential for protecting biological systems from oxidative damage. Antioxidants neutralize reactive oxygen species (ROS), prevent lipid, protein, and DNA damage, and support overall cellular homeostasis [3–5]. Several natural and synthetic compounds, including vitamins C and E, glutathione, and polyphenols, have been described as potent antioxidants that contribute to the prevention of metabolic and inflammatory disorders [6]. In addition to antioxidant properties, hydrazide–hydrazone derivatives display broad biological potential, including antimicrobial, antitubercular, antidepressant, antifungal, antiviral, antiparasitic, and anti-inflammatory effects [7]. Clinically used drugs such as isoniazid, isocarboxazid, and nitrofurazone contain hydrazide–hydrazone fragments, further demonstrating their pharmacological importance (Fig. 1) [8]. Recent studies also highlight that compounds bearing hydrazide–hydrazone moieties can effectively scavenge free radicals and mitigate oxidative stress [9]. Oxidative stress arises from an imbalance between ROS production and antioxidant defense mechanisms, and is implicated in the progression of inflammation, cancer, neurodegenerative disorders, and other chronic diseases [10, 11]. Antioxidants play a vital role in maintaining redox balance by deactivating ROS and protecting cellular integrity [12]. Inflammation is closely linked with oxidative stress, and the cyclooxygenase-2 (COX-2) enzyme is a key mediator in inflammatory pathways, catalyzing prostaglandin formation. Selective COX-2 inhibitors have demonstrated anti-inflammatory efficacy with fewer gastrointestinal side effects than traditional NSAIDs, emphasizing their therapeutic value [13]. Heterocyclic structures, such as 1,2,4-triazoles and 1,3,4-oxadiazoles, are widely recognized in drug discovery for their diverse biological activities, including antimicrobial, anticancer, anti-inflammatory, analgesic, and antioxidant properties [14–16]. Their incorporation into hydrazide–hydrazone frameworks provide an attractive strategy for developing molecules with enhanced pharmacological potential. Based on these considerations, the present study focuses on the design, synthesis, and characterization of new hydrazide–hydrazone derivatives fused with triazole and oxadiazole motifs. The antioxidant activity of the synthesized compounds was evaluated using the DPPH assay, and structural features were supported by spectroscopic analyses. In complement to the in-vitro antioxidant evaluation, molecular docking studies were conducted using the Keap1–Nrf2 protein complex as the target to assess whether the synthesized compounds could modulate. The Keap1–Nrf2 pathway is a key cellular defense mechanism against oxidative stress. Keap1 binds Nrf2 under basal conditions, promoting its degradation; however, oxidative stimuli allow Nrf2 to dissociate, enter the nucleus, and activate antioxidant gene transcription. Therefore, inhibitors of Keap1–Nrf2 binding can enhance endogenous antioxidant responses. In this study, docking was performed against the Keap1–Nrf2 interface to evaluate whether the synthesized compounds may exert antioxidant effects not only through direct radical scavenging but also via potential activation of this pathway. This study aims to identify promising heterocyclic candidates with potential antioxidant and anti-inflammatory relevance. In view of the growing interest in hydrazide–hydrazone and heterocyclic derivatives as bioactive small molecules, the present study focuses on the design and synthesis of a new series of nine compounds (**MI-1–MI-9**) incorporating pyrazole, isoindolinone, carbothioamide, triazole-thione, oxadiazole, thiazole, benzothiazole, and indole frameworks. These scaffolds were selected based on their well-recognized antioxidant potential and structural versatility in medicinal chemistry. The synthesized compounds were evaluated for their antioxidant activity using the DPPH free-radical-scavenging assay. In addition, molecular docking studies were performed to predict the interaction affinity and potential binding modes of the designed molecules with oxidative-stress-related protein targets. Density functional theory (DFT) calculations were also conducted to analyze their electronic properties, stability parameters, and structure–activity relationships. This combined synthetic, biological, and computational approach aims to identify new antioxidant candidates with promising molecular characteristics.

##  Experimental

### Synthesis of (3,5-Dimethyl-1 H-pyrazol-1-yl) (4-nitrophenyl) methanone (MI-1)

A suspension of 4-nitrobenzohydrazide (**1**) (1 mmol) and acetylacetone (**2**) (1 mmol) in absolute ethanol (20 mL) was stirred, followed by the addition of one drop of glacial acetic acid as a catalyst. The reaction mixture was heated to reflux and maintained under these conditions for 5 h. After completion, the mixture was allowed to cool to room temperature and subsequently poured onto crushed ice. The resulting solid precipitate was collected by vacuum filtration, washed thoroughly with cold distilled water, and air-dried. The crude product was purified by recrystallization from ethanol to afford compound **MI-1**.

white crystalline solid. Yield = 89%, m.p. = 98–100 °C. IR (KBr): *ν*_*max*_ 1678(C = O), 1552 (NO_2)_ 1347 (NO_2_) cm^1^. ^1^H NMR (DMSO*-d*_*6*_): *δ* 3.00 (s, 3 H, CH_3_), 3.02 (s, 3 H, CH_3_), 7.61 (m, 3 H, Ar-H), ^13^C NMR (DMSO-*d*_6_): *δ* 27.6, 27.7, 125.4 (3 C), 129.8 (3 C), 130.2, 135.0, 137.7, 142.8 ppm. Mass analysis (*m/z*, %): 245 (M+, 37), 192 (100), 105 (72),66 (50), 51(85), 43 (66). Analysis calcd. For: C_12_H_11_N_3_O_3_ (245.24). Calculated: C, 58.77; H, 4.52; N, 17.13. Found: C, 58.99; H, 4.39; N, 17.18%.

### Synthesis of 5-Methyl-2-(4-nitrobenzoyl)-2,4-dihydro-3 H-pyrazol-3-one (MI-2)

A mixture of 4-nitrobenzohydrazide **(1)** (1 mmol) and ethyl acetoacetate **(2)** (1 mmol) in **glacial acetic acid** (20 mL) was transferred to a 100 mL round-bottom flask and stirred. The reaction mixture was heated under reflux for **3 h**. The progress of the reaction was monitored by TLC. Upon completion, the mixture was allowed to cool to room temperature and then poured slowly onto crushed ice. The resulting precipitate was collected by vacuum filtration, washed thoroughly with cold distilled water to remove residual acetic acid, and air-dried. The crude product was recrystallized from dilute ethanol to afford **compound MI-2**.

Color golden-white solid. Yield = 93%, m.p. = 58–60 °C. IR (KBr): *ν*_*max*_ 2290 (CH_2_-CH_3_), 1701 (C = O), 1521 (NO_2)_ 1345 (NO_2_) cm^1^. ^1^H NMR (DMSO*-d*_*6*_): *δ* 1.35 (t, *J* = 7.20 Hz, 3 H, CH_2_), 2.51 (s, *J* = 8.40 Hz, 2 H, CH_2_), 8.20 (d, *J* = 8.80 Hz, 2 H, Ar-H), 8.36 (d, *J* = 8.80 Hz, 2 H, Ar-H).^13^C NMR (DMSO-*d*_6_): *δ* 14.5, 62.1 ,124.3 (3 C), 131.0 (3 C), 135.7, 150.6 ,164.7 ppm .Mass analysis (*m/z*, %): 247 (M+, 34), 205 (89), 172 (53),115 (100), 109(68), 96 (69), Analysis calcd. For: C_11_H_9_N_3_O_4_ (247.21). Calculated: C, 53.44; H, 3.67; N, 17.00. Found: C, 53.66; H, 3.54; N, 17.05%.

### Synthesis of 4-Nitro-(3-oxo-2,3,3a,7a-tetrahydro-1 H-isoindol-1-ylidene) benzo Hydrazide (MI-3)

A mixture of 4-nitrobenzohydrazide (**1**) (1mmol) and isatin **(4)** (1mmol) in ethanol (20 mL) was stirred in a 50 mL round-bottom flask, followed by the addition of KOH (1 mmol). The reaction mixture was heated under reflux for **4 h**, with progress monitored by TLC. Upon completion, the mixture was cooled to room temperature and poured onto crushed ice. The resulting precipitate collected by vacuum filtration, washed with cold water, and air-dried. The crude product was purified by recrystallization from ethanol to afford **compound MI-3**.

Orange crystalline solid. Yield = 96%, m.p. = 230–232 °C. IR (KBr): *ν*_*max*_ 3223(N-H), 3100 (N-H), 3055 (CH = C), 1718 (C = O), 1671 (C = O), 1545 (NO_2_), and 1339 (NO_2_) cm^1^. ^1^H NMR (DMSO*-d*_*6*_): *δ* 6.98 (d, *J* = 7.60 Hz, 1H, Ar-CH), 7.13 (t, *J* = 7.60 Hz, 1H, Ar-H), 7.42 (t, *J* = 8.00 Hz, 1H, Ar-H), 7.62 (s, 1H, N-H), 8.14 (d, *J* = 8.40 Hz, 2 H, Ar-H), 8.39 (d, *J* = 8.80 Hz, 2 H, Ar-H), 8.20 (s, 1H, C-H), 8.14 (d, *J* = 8.80 Hz, 2 H, Ar-H), 8.45 (d, *J* = 8.80 Hz, 2 H, Ar-H)^13^C NMR (DMSO-*d*_6_): *δ* 111.8 (3 C), 120.0 (2 C), 121.7, 123.3, 124.7, 129.5, 132.7, 138.1, 143.2 (3 C), 150.2, 163.4 pp. Mass analysis (*m/z*, %): 312 (M+, 19), 309 (80), 105 (72),70(79), 67(75), 45(61). Analysis calcd. For: C_15_H_12_N_4_O_4_ (312.29). Calculated: C, 57.69; H, 3.87; N, 17.94. Found: C, 57.91; H, 3.74; N, 17.99%.

### Synthesis of 2-(4-Nitrobenzoyl)-N-phenylhydrazine-1-carbothioamide (MI-4)

A stirred solution of 4-nitrobenzohydrazide (**1**) (1 mmol) and phenyl isothiocyanate (**5**) (1 mmol) in ethanol (20 mL) was heated under reflux for 3 h. After cooling to room temperature, the resulting precipitate was collected by vacuum filtration, washed with cold ethanol, and dried in vacuo. The crude solid was purified by recrystallization from dilute ethanol to afford **MI-4** as a crystalline product.

Yellow crystalline color. Yield = 91%, m.p. = 178–180 °C. IR (KBr): *ν*_*max*_ 3300(N-H), 3200(N-H), 3100(N-H), 1595(C = O), 1543 (NO_2)_ 1371 (NO_2_) cm^1^. ^1^H NMR (DMSO*-d*_*6*_): *δ* 7.11 (d, 2 H, *J* 7.00 Hz, Ar-H), 7.32 (s, 3 H, Ar-H), 7.63 (s, 2 H, Ar-H), 11.0 (s, 1H, N-H). Mass analysis (*m/z*, %): 316 (M+, 23), 166 (75), 157 (100), 144 (97). Analysis calcd. For: C_14_H_12_N_4_O_3_S (316.34). Calculated: C, 53.16; H, 3.82; N, 17.71; Found: C, 53.38; H, 3.69; N, 17.76%.

### Synthesis of 5-(4-Nitrophenyl)-4-phenyl-2,4-dihydro-3 H-1,2,4-triazole-3-thione (MI-5)

A mixture of **MI-4** (1 mmol) and aqueous KOH (1 mmol) was stirred and heated under reflux for 4 h. Upon completion, the reaction mixture was cooled to room temperature and acidified carefully with dilute HCl to induce precipitation. The resulting solid was collected by vacuum filtration, washed with cold water, and air-dried. The crude product was purified by recrystallization from ethanol to afford **MI-5.**

White color. Yield = 95%, m.p. = 278–280 °C. IR (KBr): *ν*_*max*_ 3563 (N-H), 1659 (C = O) cm^1^. ^1^H NMR (DMSO*-d*_*6*_): *δ* 7.39 (d, *J* = 4.00 Hz, 3 H, Ar-H ), 7.50 (t, *J* = 1.50 Hz, 3 H, Ar-H), 7.54 (d, *J* = 8.50 Hz, 2 H, Ar-H), 8.17 (d, *J* = 8.50 Hz, 2 H, Ar-H), 14.35 (s, 1H, S-H) ^13^C NMR (DMSO-*d*_6_): *δ* 121.7(2 C), 123.1, 124.6, 125.0, 128.4 (2 C), 128.9, 137.8, 138.6, 187.2, 187.8 ppm. Mass analysis (*m/z*, %):298 (M+, 27), 244 (56), 234 (53), 181 (55), 166 (75), 92 (57), 69 (100). Analysis calcd. For: C_14_H_10_N_4_O_2_S (298.32). Calculated: C, 56.37; H, 3.38; N, 18.78; Found: C, 56.59; H, 3.25; N, 18.83%.

### Synthesis of 5-(4-Nitrophenyl)-1,3,4-oxadiazole-2-thiol (MI-6)

A mixture of 4-nitrobenzohydrazide (**1**) (1 mmol) and carbon disulfide (1 mmol) in ethanol (20 mL) was stirred, and KOH was added. The reaction mixture was heated under reflux for 5 h. After cooling to room temperature, the resulting precipitate was collected by vacuum filtration, washed with cold ethanol, and air-dried. The crude product was purified by recrystallization from ethanol to afford **MI-6**.

orange color. Yield = 96%, m.p. = 218–230 °C. IR (KBr): *ν*_*max*_ 1545 (NO_2)_ 1339 (NO_2_) cm^1^. ^1^H NMR (DMSO*-d*_*6*_): *δ* 6.21 (s, 2 H, CH_2_), 7.18 (d, *J* = 8.40 Hz, 1H, Ar-H), 7.58 (d, *J* = 8.40 Hz, 1H, Ar-H), 7.68 (s, 1H, Ar-H), 8.14 (d, *J* = 8.40 Hz, 2 H, Ar-H), 8.39 (d, *J* = 8.80 Hz, 2 H, Ar-H), 8.20 (s, 1H, C-H), 12.03 (s, 1H, N-H) ^13^C NMR (DMSO-*d*_6_): *δ* 124.6, 1125.7(3 C), 130.5, 147.3, 159.6, 181.5, ppm. Analysis calcd. For: C_8_H_5_N_3_O_3_S (223.21). Calculated: C, 43.05; H, 2.26; N, 18.83. Found: C, 43.27; H, 2.13; N, 18.88%.

### Synthesis of 2-((5-(4-Nitrophenyl)-1,3,4-oxadiazol-2-yl) thiol)-N-(thiazol-2-yl) acetamide (MI-7)

A mixture of **MI-6** (1 mmol) and 2-chloro-*N*-(1,3-thiazol-2-yl) acetamide **(7)** (10 mmol) in dry acetone (20 mL) was stirred, and K_2_CO_3_ (1 mmol) was added. The reaction mixture was heated under reflux for 5 h. Upon completion, the mixture was cooled to room temperature, and the resulting was collected by vacuum filtration, washed with cold acetone, and air-dried. The crude product was purified by recrystallization from dilute ethanol to afford **MI-7**.

pale-yellow powder. Yield = 98%, m.p. = 216–218 °C. IR (KBr): *ν*_*max*_ 3100 (N-H), 1694 (C = O), 1516 (NO_2)_ 1343 (NO_2_) cm^1^. ^1^H NMR (DMSO*-d*_*6*_): *δ* 4.29 (s, 2 H, CH_2_), 6.93 (d, *J* = 8.00 Hz, 1H, Ar-H), 7.32 (d, *J* = 8.00 Hz, 1H, Ar-H), 8.21 (d, *J* = 9.00 Hz, 2 H, Ar-H), 8.38 (d, *J* = 8.50 Hz, 2 H, Ar-H) ^13^C NMR (DMSO-*d*_6_): *δ* 110.0, 124.6 (3 C), 127.7 (3 C), 128.7, 136.8, 149.0, 163.3, 166.5, 167.8 ppm. Mass analysis (*m/z*, %): 363 (M+, 11), 357 (63), 343 (68), 254 (81), 220 (52), 178 (77),152(100),145(92),131(93), Analysis calcd. For: C_13_H_9_N_5_O_4_S_2_(363.37). Calculated: C, 42.97; H, 2.50; N, 19.27; Found: C, 43.19; H, 2.37; N, 19.32%.

###  Synthesis of N-(benzo[d]thiazol-2-yl)-2-((5-(4-nitrophenyl)-1,3,4-oxadiazol-2-yl)thio)acetamide (MI-8)

A mixture of **MI-6** (1 mmol) and *N*-benzothiazol-2-yl-2-chloroacetamide **(8)** (1 mmol) in dry acetone (20 mL) was stirred, and K_2_CO_3_ (1 mmol) was added. The reaction mixture was heated under reflux for 5 h. After cooling to room temperature, the resulting precipitate was collected by vacuum filtration, washed with cold acetone, and air-dried. The crude product was purified by recrystallization from dilute ethanol to afford **MI-8**.

brown powder. Yield = 95%, m.p. = 210–212 °C. IR (KBr): *ν*_*max*_ 3563 (N-H), 1692 (C = O), 1509 (NO_2)_ 1317 (NO_2_) cm^1^. ^1^H NMR (DMSO*-d*_*6*_): *δ* 4.21 (s, 2 H, CH_2_), 6.74 (d, *J* = 8.40 Hz, 1H, Ar-H), 7.08 (d, *J* = 8.40 Hz, 2 H, Ar-H), 7.23 (d, *J* = 8.40 Hz, 1H, Ar-H), 7.39 (d, *J* = 8.40 Hz, 2 H, Ar-H), 8.39 (m, *J* = 8.80 Hz, 3 H, Ar-H)^13^C NMR (DMSO-*d*_6_): *δ* 35.3, 113.2, 125.0 (5 C), 128.1 (3 C), 129.2, 137.3, 137.9(2 C),149.4,163.8,168.9ppm. Mass analysis (*m/z*, %): 413 (M+, 30), 406 (52), 361 (83), 326 (72), 176 (100), 108 (87), Analysis calcd. For: C_17_H_11_N_5_O_4_S_2_(413.43). Calculated: C, 49.39; H, 2.68; N, 16.94;Found: C, 49.61; H, 2.55; N, 16.99%.

### Synthesis of N-(1 H-indol-6-yl)-2-((5-(4-nitrophenyl)-1,3,4-oxadiazol-2-yl)thio)acetamide (MI-9)

A mixture of **MI-6** (1 mmol) and 1*H*-indol-6-yl) carbamic chloride **(9)** (1 mmol) in dry acetone (20 mL) was stirred, and K_2_CO_3_ (1 mmol) was added. The reaction mixture was heated under reflux for 5 h. After cooling to room temperature, the resulting precipitate was collected by vacuum filtration, washed with cold acetone, and air-dried. The crude product was purified by recrystallization from dilute ethanol to afford **MI-9**.

black powder. Yield = 80%, m.p. = above 300 °C. IR (KBr): *ν*_*max*_ 3563 (N-H), 1674 (C = O), 1525 (NO_2_), 1340 (NO_2_) cm^1^. ^1^H NMR (DMSO*-d*_*6*_): *δ* 6.21 (s, 2 H, CH_2_), 7.18 (d, *J* = 8.40 Hz, 1H, Ar-H), 7.58 (d, *J* = 8.40 Hz, 1H, Ar-H), 7.68 (s, 1H, Ar-H), 8.14 (d, *J* = 8.40 Hz, 2 H, Ar-H), 8.39 (d, *J* = 8.80 Hz, 2 H, Ar-H), 8.20 (s, 1H, C-H), 12.03 (s, 1H, N-H) ^13^C NMR (DMSO-*d*_6_): *δ* 105.68(C), 116.75 (C), 124.13 (C), 124.17 (C), 124.31 (C), 126.27 (C), 129.52 (C), 138.34 (C), 139.66 (C), 148.64 (C), 149.20 (C), 149.70 (C), 149.85(C), 149.97 (C), 151.93 (C), 151.97 (C), 161.83 (C), 164.61 (C) ppm. Mass analysis (*m/z*, %): 395 (M+, 44), 387 (51), 294(100), 59 (56), Analysis calcd. For: C_18_H_13_N_5_O_4_S (395.39). Calculated: C, 54.68; H, 3.31; N, 17.71. Found: C, 54.90; H, 3.18; N, 17.76%.

##  Molecular geometry optimization and quantum reactivity analysis

The initial structures of the synthesized compounds were constructed using Chem3D (version 16.0) and saved in (*.mol) format. Geometry optimization and subsequent quantum-chemical calculations were performed using Gaussian 09 W software [17]. Density Functional Theory (DFT) was employed using the Becke three-parameter hybrid functional combined with the Lee–Yang–Parr correlation functional (B3LYP) with the 6-311 + + G(d, p) basis set [18]. This level of theory was selected due to its widely documented balance between computational cost and accuracy in organic molecular systems. The optimized structures were confirmed as true minima by the absence of imaginary frequencies. The B3LYP/6-311 + + G(d, p) method was used to calculate total energies [19], HOMO-LUMO energy levels, energy gaps (Eg), dipole moments (µ), and global reactivity descriptors including electronegativity (χ), chemical hardness (η), softness (σ), and nucleophilicity index (Nu) [20]. The 6-311 + + G(d, p) basis set provides triple-zeta split-valence precision and incorporates polarization functions (d-orbitals on heavy atoms and p-orbitals on hydrogen), allowing improved treatment of molecular geometry and bonding. The additional diffuse functions (++) [21], applied to both heavy atoms and hydrogens, enhance accuracy for systems containing lone pairs, π-conjugation, and weak intermolecular interactions. Overall, the B3LYP/6-311 + + G(d, p) level of theory offers a robust and reliable platform for predicting the electronic structure and reactivity parameters of organic molecules in agreement with previously reported studies [22]– [23].

### DFT-Based computational procedures

The three-dimensional structures of the carbohydrazide derivatives **MI-1** to **MI-9** were constructed using GaussView 6.0 and optimized at the DFT level using Gaussian 09 W software packages [24]. Geometry optimization was performed without symmetry constraints, and frequency calculations were subsequently carried out to verify that all structures corresponded to true minima on the potential energy surface (i.e., no imaginary frequencies were observed).

Frontier molecular orbital (FMO) parameters, including HOMO and LUMO energies and the HOMO–LUMO energy gap (ΔE), were calculated to evaluate the electronic stability and potential reactivity of the compounds. Additionally, molecular electrostatic potential (MEP) maps were generated to identify electron-rich (nucleophilic) and electron-deficient (electrophilic) regions, providing insight into the possible interaction sites relevant to antioxidant behavior and molecular docking studies [6].

## Antioxidant activity using DPPH assay

The antioxidant capacity of the examined sample was assessed using the DPPH^•^ colorimetric method, with ascorbic acid serving as the standard, according to the assay described by Kitts et al. The serial dilution of each sample was conducted by combining the sample with an equal volume of methanol. A DPPH^•^ solution was produced at a concentration of 0.135 mM and combined with each sample in a serial dilution with an equal amount. Following the introduction of the DPPH^•^ solution, the samples were maintained in darkness for 30 min at ambient temperature. The absorbance of each sample was subsequently measured at 517 nm. The percentage of residual DPPH^•^ was determined using the following equation (Eq. (1)) [25]:

1 $$ {\mathrm{DPPH}}\,{\mathrm{remaining}}\,{\text{ = }}\frac{{\left[ {{\mathrm{DPPH}}} \right]_{{\mathrm{T}}} }}{{\left[ {{\mathrm{DPPH}}} \right]_{{{\text{T = 0}}}} }}\, \times 100 $$.

The percentages of remaining DPPH^•^ were graphed against the sample concentration in mg/mL using an exponential curve to determine the effective concentration, referred to as “IC_50_.” IC_50_ denotes the specific quantity of antioxidants required to reduce the initial concentration of DPPH^•^ solution by 50%. The IC_50_ values indicate an inverse correlation with the antioxidant capacity of the examined sample [26].

## Results and discussion

**Scheme 1:**
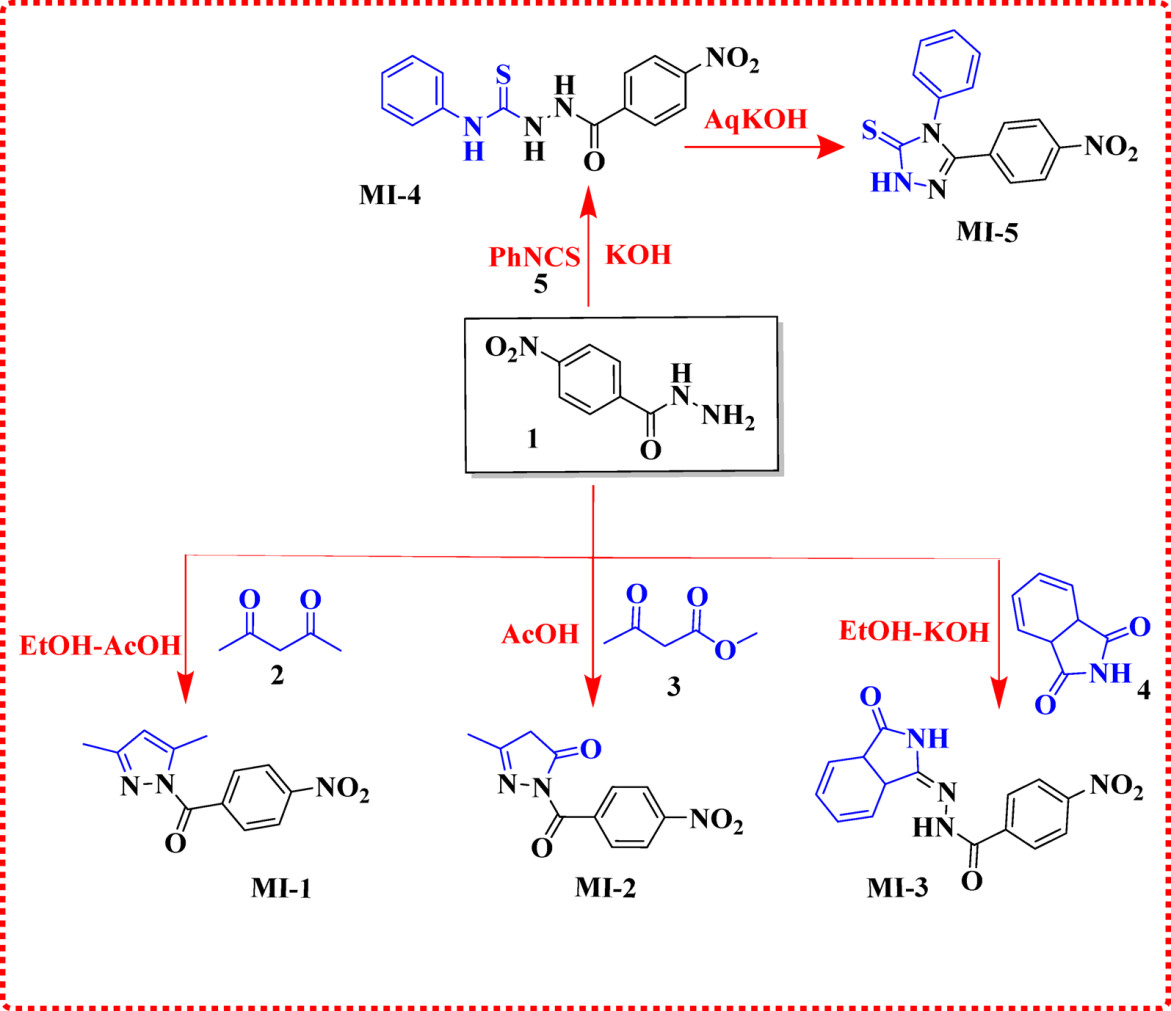
Synthesis of Hydrazide derivatives** MI-1-MI-5**

The condensation of acetylacetone (**2**) with 4-nitrobenzohydrazide in ethanol afforded the novel pyrazole derivative **MI-1**. Elemental analysis confirmed the molecular formula (MF), and the structure was validated using IR, ^1^H NMR, and ^13^C NMR spectroscopy. The IR spectrum exhibited characteristic bands corresponding to the C = O and NO_2_ groups at 1678, 1552, and 1347 cm⁻¹. In the ¹H NMR spectrum, the methyl protons (2CH_3_) appeared as two singlets at δ 3.00 and 3.02 ppm, while the corresponding ^13^C NMR signals for the methyl carbons were observed at δ 27.6 and 27.4 ppm. The novel pyrazolone derivative **MI-2** was synthesized via condensation of 4-nitrobenzohydrazide with ethyl acetoacetate (EAA) in ethanolic KOH. IR analysis revealed bands corresponding to CH_2_–CH_2_, C = O, and NO_2_ groups at 2290, 1701, 1521, and 1345 cm⁻¹. In the ¹H NMR spectrum, singlets at δ 2.03 and 3.05 ppm were assigned to CH₂ and CH₃ protons, respectively. The ^13^C NMR spectrum displayed signals at δ 14.52, 62.1, and 164.5 ppm corresponding to the carbonyl and methyl carbons. Condensation of 4-nitrobenzohydrazide with isatin in acetic acid yielded the isoindolinone hydrazide derivative **MI-3**. IR spectra showed characteristic bands for N–H, C = O, and NO_2_ groups at 3223, 3100, 1718, 1545, and 1339 cm⁻¹. The ^13^C NMR spectrum revealed carbonyl carbons at δ 163.4 ppm. The mass spectrum displayed a molecular ion peak at m/z 312 (19.86%), consistent with the calculated molecular mass of C_15_H_12_N_4_O_4_, confirming the successful formation of the target structure. The thiosemicarbazide **MI-4** exhibited IR bands corresponding to N–H, C = O, and NO_2_ groups at 3563, 1695, 1545, and 1339 cm^− 1^. In the ¹H NMR spectrum, N–H protons appeared as two singlets at δ 14.35 ppm. The molecular ion peak at m/z 298 (27.34%) matched the calculated molecular formula C_14_H_10_N_4_O_2_S, supporting the successful synthesis of the thiosemicarbazide. Cyclization of **MI-4** with KOH produced the 2,4-dihydro-1,2,4-triazole-3-thione derivative **MI-5**. IR spectra showed bands for three N–H, C = O, and NO_2_ groups at 3300, 3200, 3100, 1595, 1543, and 1371 cm⁻¹. The mass spectrum displayed a molecular ion peak at m/z 316 (23.62%), consistent with the calculated molecular mass for C_14_H_12_N_4_O_3_S, confirming the formation of the 1,2,4-triazole-3-thione product as shown in Scheme 1.

**Scheme 2:**
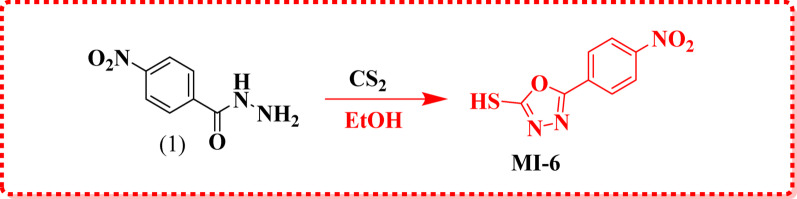
Synthesis of 1,3,4-oxadiazole thione **MI-6**

The cyclization of 4-nitrobenzohydrazide with carbon disulfide in the presence of potassium hydroxide afforded the 5-mercapto-1,3,4-oxadiazole derivative **MI-6** (Scheme 2). The FT-IR spectrum of **MI-6** displayed characteristic NO_2_ stretching bands at 1545 and 1339 cm^− 1^. In the ¹H NMR spectrum, a singlet signal observed at δ 11.24 ppm confirmed the presence of the thiol (–SH) proton. The structure was further supported by mass spectrometry, where the molecular ion peak appeared at m/z = 223 (16.32%), consistent with the calculated molecular weight for C_8_H_5_N_3_O_3_S, thereby confirming the molecular formula.

**Scheme 3:**
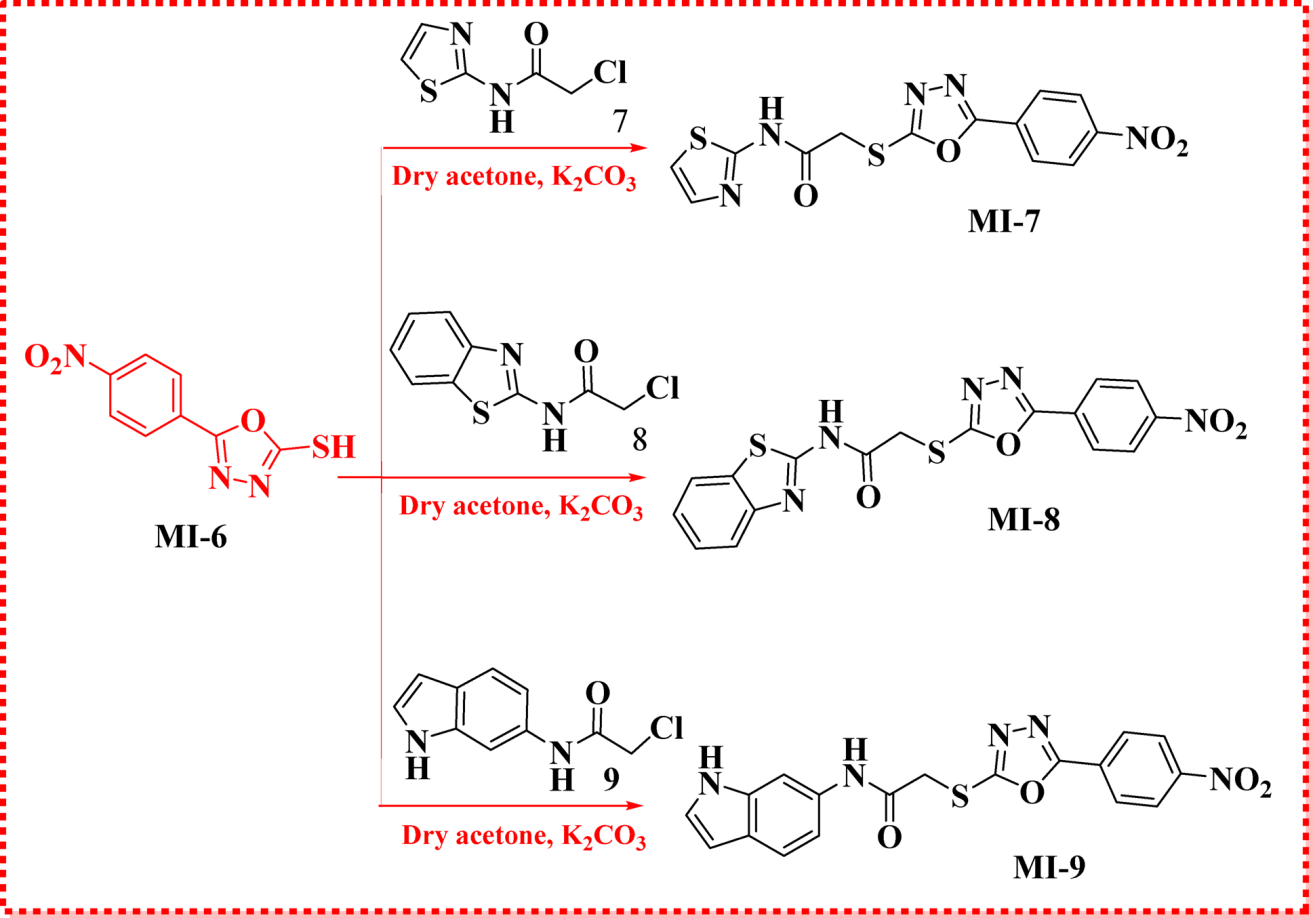
Synthesis of ((1,3,4-oxadiazol-2-yl) thio) acetamide derivatives **MI-7-9**

The 5-mercapto-1,3,4-oxadiazole core was further functionalized via condensation with various acetamides, namely 2-chloro-*N*-(1,3-thiazol-2-yl)acetamide **(7)**, *N*-benzothiazol-2-yl-2-chloroacetamide **(8)**, and (1*H*-indol-6-yl)carbamic chloride **(9)**, affording the derivatives **MI-7**,** MI-8**,** and MI-9**, respectively [17–19]. The molecular formulas of these compounds were confirmed by elemental analysis, while their structures were elucidated using IR, ¹H NMR, and ^13^C NMR spectroscopy. For **MI-7**, the FT-IR spectrum displayed characteristic bands for N–H, C = O, and NO_2_ groups at 3100, 1644, 1516, and 1343 cm⁻¹. In the ¹H NMR spectrum, a singlet at δ 4.12 ppm was assigned to the CH_2_ protons, while the ^13^C NMR spectrum showed the carbonyl carbon signal at δ 167.8 ppm. Mass spectrometry revealed a molecular ion peak at m/z 363 (11.05%), consistent with the calculated molecular formula **C**_**13**_**H**_**9**_**N**_**5**_**O**_**4**_**S**_**2**_. For **MI-8**, the IR spectrum exhibited absorption bands at 3563, 1692, 1509, and 1317 cm⁻¹, corresponding to N–H, C = O, and NO_2_ functionalities. The ^1^H NMR spectrum displayed a singlet at δ 2.40 ppm for the CH_2_ protons, while the ^13^C NMR signals for the CH_2_ and carbonyl carbons were observed at δ 35.30 and 168.9 ppm, respectively. The mass spectrum showed a molecular ion peak at m/z 413 (30.27%), supporting the molecular formula **C**_**17**_**H**_**11**_**N**_**5**_**O**_**4**_**S**_**2**_. For **MI-9**, characteristic IR bands for CH_2_–CH_3_ and C = O groups were observed at 2290, 1701 and 1521 cm⁻¹. The ¹H NMR spectrum exhibited a singlet at δ 4.44 ppm corresponding to the CH₂ protons. Mass spectrometry revealed a molecular ion peak at m/z 395 (44.42%), consistent with the proposed molecular formula **C**_**18**_**H**_**13**_**N**_**5**_**O**_**4**_**S**. The synthetic transformations are summarized in Scheme 3.

## Frontier molecular orbital (FMO) analysis

FMO calculations demonstrated that all MI derivatives (**MI-1–MI-9**) possessed stable energy-minimized structures with no imaginary frequencies. The compounds showed variations in HOMO and LUMO energies, indicating differing electron-donating/accepting capacities and potential antioxidant behavior. Molecules with higher HOMO levels and lower HOMO–LUMO gaps (ΔE) are expected to exhibit enhanced electron-transfer ability and improved radical-scavenging potential [27]. Table 1 presents the computed global reactivity descriptors, including electronegativity (χ), chemical potential (µ), global hardness (η), electrophilicity index (ω), global softness (σ), and nucleophilicity index (Nu), all derived according to Eqs. (2, 3, 4, 5, 6, 7) [28]– [12].

The following global reactivity descriptors were calculated according to established density functional theory (DFT) equations:

2 $$ \chi {\mkern 1mu} = - \frac{1}{2}{\mathrm{(}}E_{{HOMO}} \,{\text{ + }}\,E_{{LUMO}} {\mathrm{)}} $$

3 $$ \mu \, = - \,\chi = \,\frac{1}{2}\,\left( {{\mathrm{E}}_{{{\mathrm{HOMO}}}} - {\mathrm{E}}_{{{\mathrm{LUMO}}}} } \right) $$

4 $$ \eta \, = \,\frac{1}{2}\,\left( {{\mathrm{E}}_{{{\mathrm{HOMO}}}} - {\mathrm{E}}_{{{\mathrm{LUMO}}}} } \right) $$

5 $$ \omega {\mkern 1mu} {\text{ = }}{\mkern 1mu} \frac{{\mu {\mathrm{2}}}}{{{\mathrm{2}}\eta }} $$

6 $$ \sigma {\mkern 1mu} \frac{1}{{2\eta }} $$

7 $$ Nu\, = \,\frac{1}{\omega } $$

The enhancement of antioxidant activity in a molecule is frequently linked to a diminished band gap energy between its (HOMO) and (LUMO), or to the eigenvalue of the HOMO. This represents a body of established evidence. In pursuit of this particular objective, we meticulously assessed the quantum technological attributes inherent to each molecule. The results are delineated in Table 1. The orbitals of the highest occupied molecular orbital and the lowest unoccupied molecular orbital were used to analyze how their wavefunctions are spread out and how reactive they are. Table 1 shows that changing the substituents affected the band gap energies and reactivity of the compounds from **MI-1** to **MI-9**. This procedure was applicable to all the compounds.

**Table 1:** The quantum chemical descriptors for **MI-1-9**

Compounds	E_HOMO_	E_LUMO_	E_gap_	Χ	µ	η	σ	ω	Nu
**MI-1**	-6.80	-2.95	3.85	4.87	-4.87	1.92	1.04	6.17	0.16
**MI-2**	-6.53	-3.26	3.27	4.89	-4.89	1.63	1.22	7.33	0.13
**MI-3**	-5.44	-2.72	2.72	4.08	-4.08	1.36	1.47	6.12	0.16
**MI-4**	-6.25	-2.99	3.26	4.62	-4.62	1.62	1.23	6.58	0.15
**MI-5**	-5.98	-2.93	3.05	4.45	-4.45	1.52	1.31	6.51	0.14
**MI-6**	-7.07	-3.07	4.02	5.07	-5.07	2.01	0.99	6.39	0.15
**MI-7**	-6.24	-3.10	3.14	4.67	-4.67	1.57	1.27	6.94	0.14
**MI-8**	-6.47	-3.07	3.40	4.77	-4.77	1.70	1.17	6.69	0.14
**MI-9**	-6.53	-2.99	3.54	4.76	-4.76	1.77	1.29	6.40	0.15

**Fig. 2:**
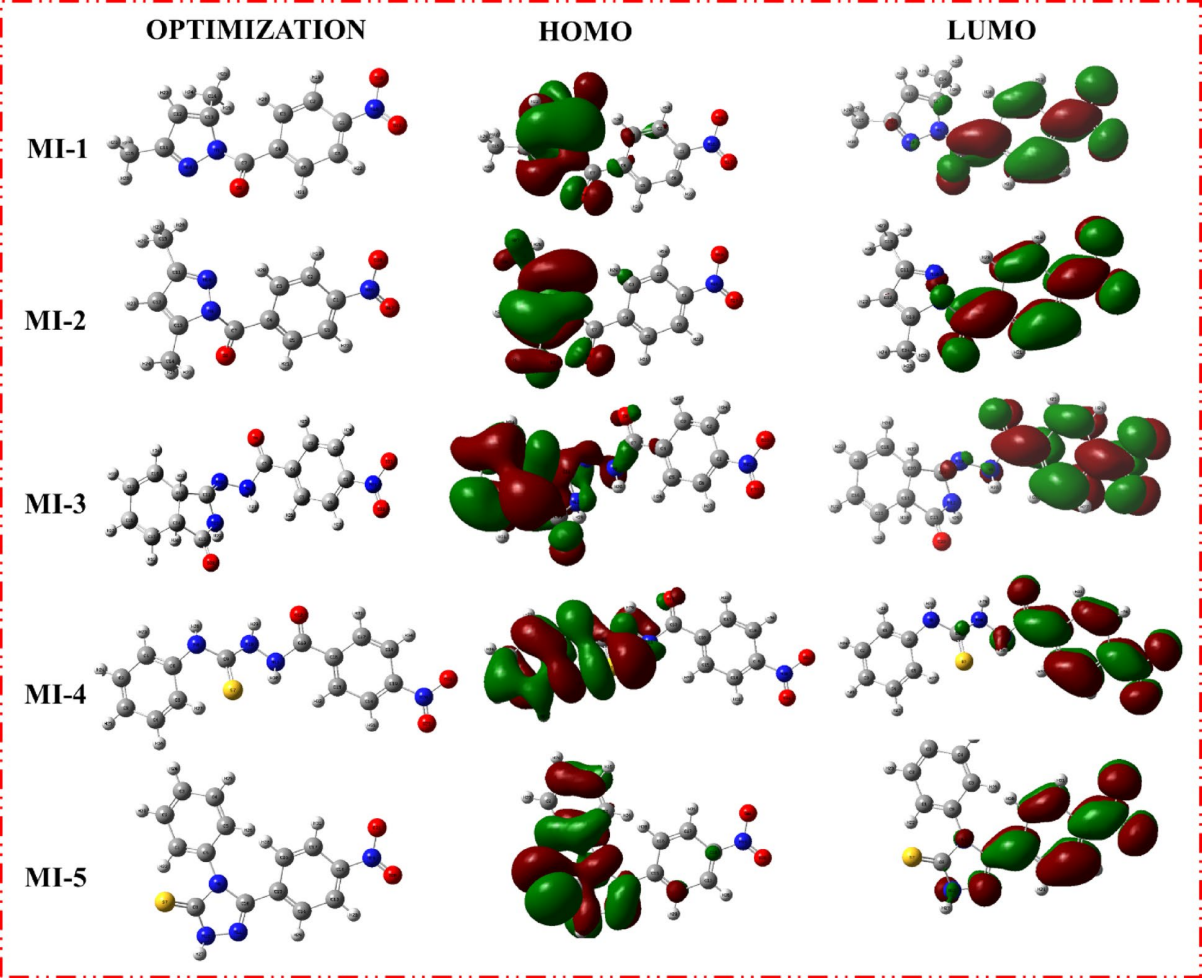
Optimization geometries, LUMOs geometries and HOMOs geometries for sensitizers **MI-1** to **MI-5**

The frontier molecular orbital (FMO) analysis of compounds **MI-1** to **MI-9** revealed significant variations in their electronic structures, which directly influenced their stability, reactivity, and antioxidant potential. The HOMO energies ranged from − 7.07 eV (**MI-6**) to − 5.44 eV **(MI-3**), while the LUMO energies varied between − 3.26 eV (**MI-2)** and − 2.72 eV (MI-3), giving band gap (E_gap_) values of 2.72–4.02 eV. Notably, **MI-3** displayed the lowest energy gap (2.72 eV), indicating a highly delocalized electron density and greater chemical reactivity, while **MI-6** exhibited the highest gap (4.02 eV), suggesting enhanced molecular stability due to the strong electron-withdrawing nature of its oxadiazole-thione moiety. Electronegativity (χ) values ranged from 4.08 eV (**MI-3**) to 5.07 eV (**MI-6**), and chemical potentials (µ) were consistently negative, reflecting a strong tendency for electron acceptance. Hardness (η) values were lowest for **MI-3** (1.36 eV), designating it as the softest and most polarizable compound, while **MI-6** had the highest hardness (2.01 eV), correlating with its higher stability. Softness (σ) and electrophilicity (ω) indices further emphasized these trends, with **MI-2** showing the highest electrophilicity (7.33 eV), making it a strong electron acceptor. The reciprocal electrophilicity (1/ω) index was highest for **MI-1** (0.17 eV ^− 1^), reflecting its greater electron-donating capability. These findings suggest that compounds with lower energy gaps and higher softness, such as **MI-3 and MI-5**, are more reactive and likely to exhibit enhanced radical scavenging activity, whereas **MI-6**, with its high electronegativity and electrophilicity, is well-suited for interactions with oxidative species. The (FMO) maps of **MI-1 to MI-9** (Figs.2 and3) reveal distinct electronic distributions that influence their antioxidant behavior. In most derivatives, the **HOMO** orbitals are primarily delocalized over the hydrazide–hydrazone backbone and adjacent heteroaryl moieties, indicating strong electron-donating potential, whereas the **LUMO** orbitals are mainly concentrated on the nitro and oxadiazole/triazole groups, reflecting their electron-accepting nature [29]. This spatial separation facilitates intramolecular charge transfer, which is particularly pronounced in **MI-3 and MI-5**, where extended delocalization over the conjugated heterocyclic framework contributes to their reduced band gaps **(2.72–3.05 eV)** and enhanced reactivity. Conversely, **MI-6** exhibits more localized orbital density with a higher energy gap **(4.02 eV)**, correlating with its lower reactivity. These electronic distributions highlight that greater HOMO delocalization and HOMO-LUMO overlap, as seen in **MI-3** and **MI-4**, promote efficient electron transfer and radical quenching, aligning with their superior antioxidant activity in the DPPH assay. (FMO) analysis demonstrated clear differences in the electronic profiles of the **MI** derivatives. **MI-3** exhibited the smallest HOMO–LUMO energy gap (2.72 eV), indicating the highest electronic polarizability and reactivity, while **MI-6** showed the highest gap (4.02 eV), reflecting greater stability due to its electron-withdrawing oxadiazole-thione core. Compounds with lower Egap values (**MI-3**,** MI-5**) possess superior electron-transfer ability, supporting their enhanced radical-scavenging behavior. In contrast, **MI-6** exhibited the highest electronegativity (χ = 5.07 eV) and hardness (η = 2.01 eV), suggesting strong electron-acceptance capability and reduced reactivity. Global reactivity indices reinforced these trends. **MI-2** displayed the highest electrophilicity (ω = 7.33 eV), indicating strong affinity toward electron-rich radical species, whereas **MI-1** showed the highest reciprocal electrophilicity (*Nu* = 0.17 eV ^− 1^), consistent with stronger electron-donation ability. FMO visualizations confirmed that HOMO densities are predominantly distributed over the hydrazide-hydrazone and heteroaryl systems, enabling efficient electron donation, while LUMO orbitals localize around electron-deficient nitro and heterocyclic fragments, promoting intramolecular charge transfer. This orbital configuration is most pronounced in **MI-3** and **MI-5**, aligning with their superior predicted antioxidant reactivity [30].

**Fig. 3:**
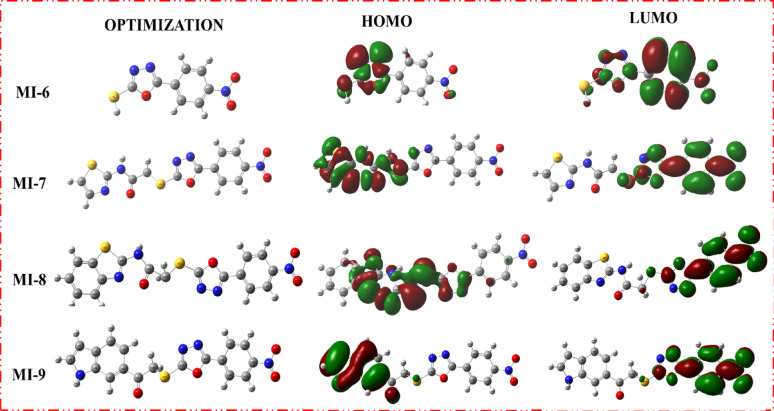
Optimization geometries, LUMOs geometries and HOMOs geometries for sensitizers **MI-6** to **MI-9**

### Molecular electrostatic potential (MEP) surface analysis

**Fig. 4:**
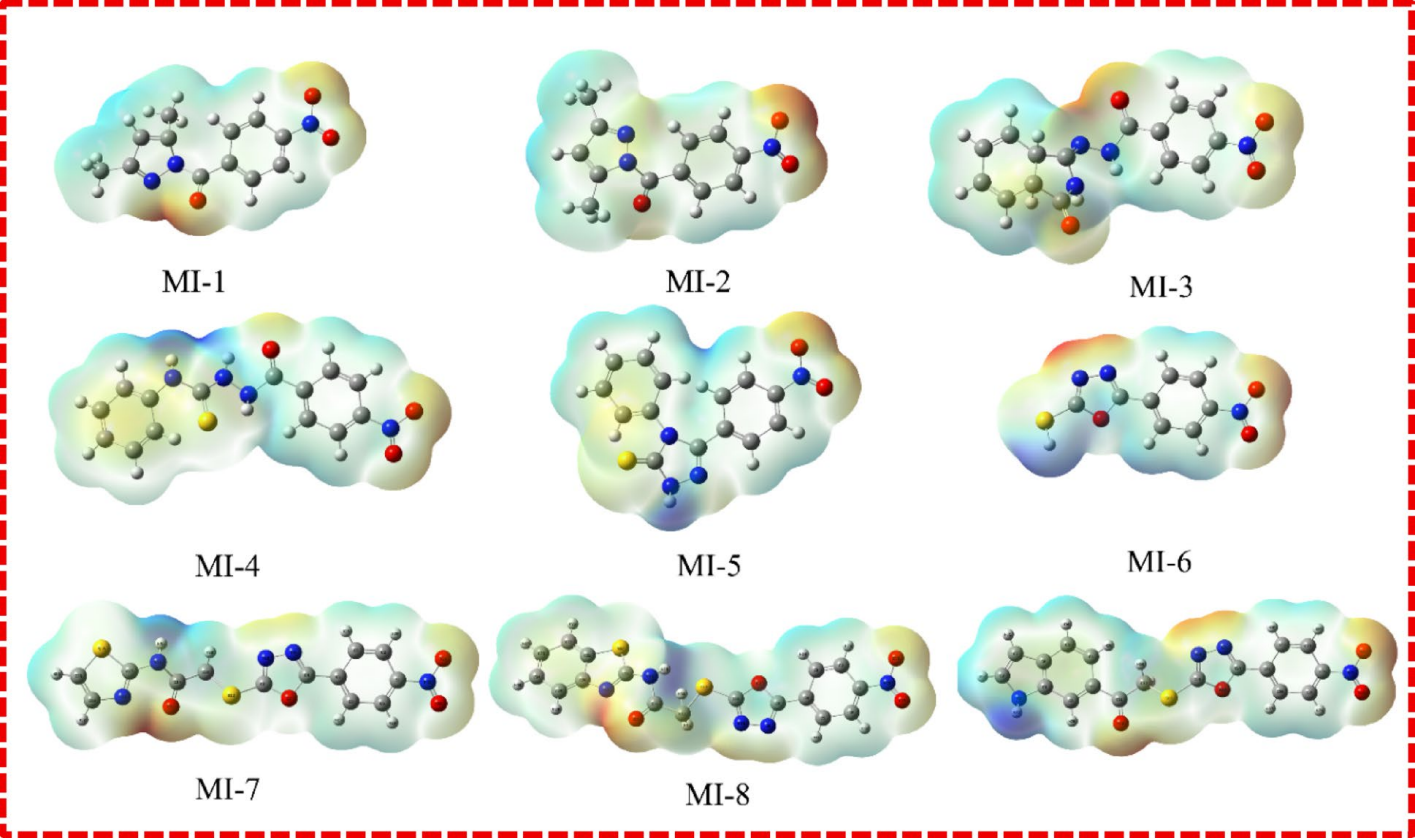
The molecular electrostatic potential of the compounds **MI-1** to **MI-9**

Molecular electrostatic potential (MEP) mapping was employed to visualize the charge distribution and identify the electrophilic and nucleophilic reactive sites of compounds **MI-1** to **MI-9**. The MEP surfaces are presented in Fig. 4. In these maps, electron-rich (electronegative) regions appear in red–yellow [31], indicating susceptibility to electrophilic attack and serving as hydrogen-bond acceptor sites, while electron-deficient (electropositive) regions are shown in blue, representing potential nucleophilic attack and hydrogen-bond donor sites. Compounds **MI-1** and **MI-2**, which possess pyrazole and pyrazolone cores, demonstrated pronounced negative potential around the carbonyl oxygen atoms and the nitro group. These electron-rich sites may facilitate hydrogen-bonding interactions and allow for effective scavenging of electrophilic free radicals, explaining the moderate antioxidant response observed for these molecules. Their positive regions, primarily localized over the pyrazole N–H and adjacent C–H centers, further support their ability to participate in proton-donation and radical stabilization mechanisms. For **MI-3**, the isoindolinone framework displayed intense negative potential zones on the two carbonyl functionalities and nitro substituent, suggesting strong electron-donating capacity and affinity toward electrophilic radical species. **MI-4** exhibited a similar pattern, with an additional electronegative hotspot around the thioamide sulfur, enhancing nucleophilic character and providing multiple interaction centers. The sulfur-associated red zone in **MI-4** correlates with its increased reactivity and potential to chelate or interact with redox-active species. Compounds **MI-5** to **MI-9**, bearing triazole and oxadiazole heterocycles, displayed even more intense negative electrostatic distributions across heteroatoms (N, O, S) and nitro groups. **MI-5** showed broad red potential around sulfur and carbonyl oxygen sites, indicative of strong radical-scavenging capacity. **MI-6** to **MI-9** exhibited extensive electron-rich zones on their oxadiazole sulfur and nitro-phenyl moieties, with **MI-7** to **MI-9** further showing electropositive halos near amide N–H sites, enhancing hydrogen-bond interactions. Notably, **MI-7**,** MI-8**, and **MI-9** presented the highest polarity contrast, reflecting enhanced capability to stabilize transition states and interact with biological targets. These observations correlate well with their superior antioxidant behavior and docking results.

## Antioxidant activity (DPPH Assay)

**Fig. 5:**
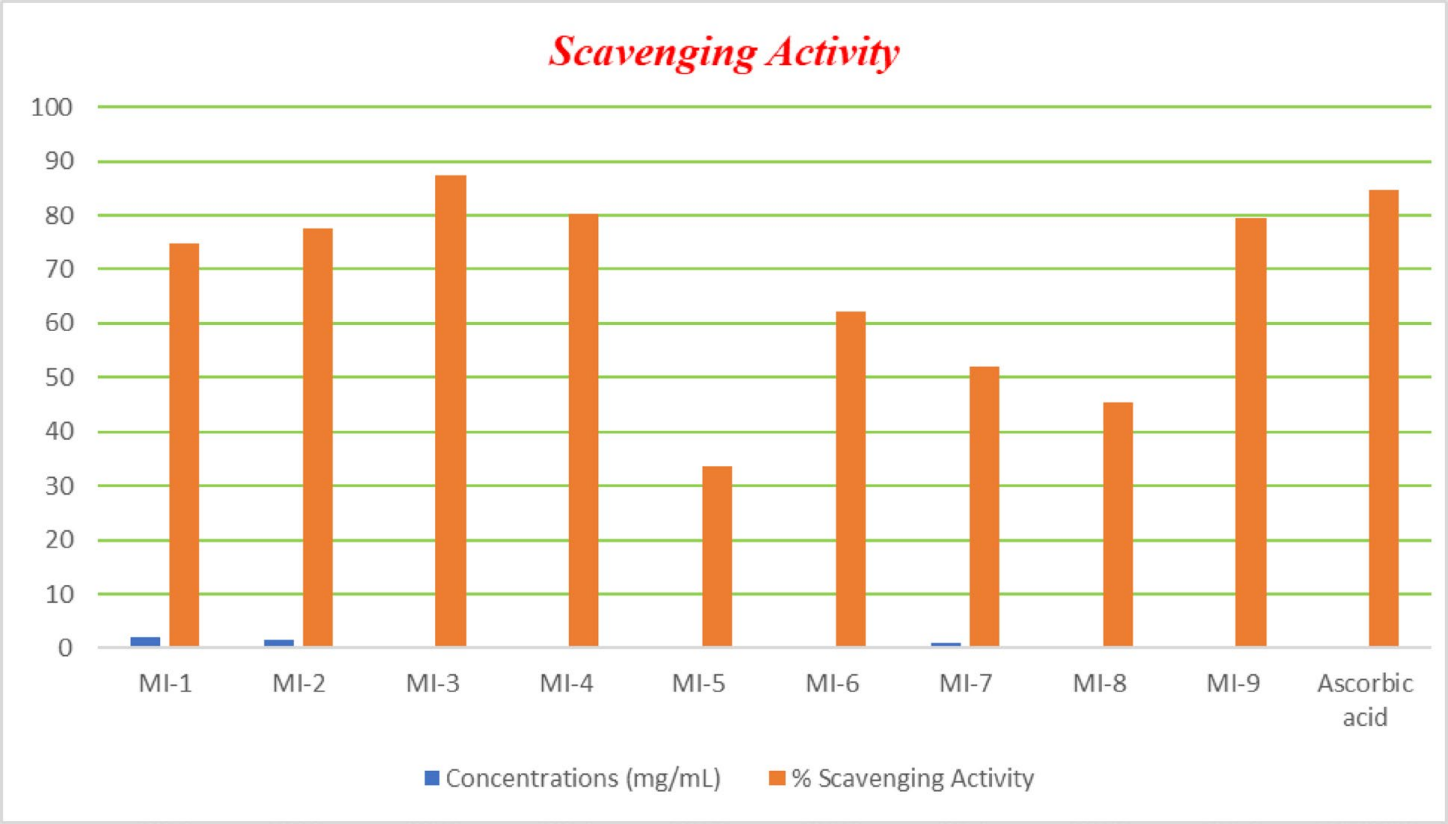
For the percentage of free radical scavenging activity, dose-responsive curves were plotted

**Fig. 6:**
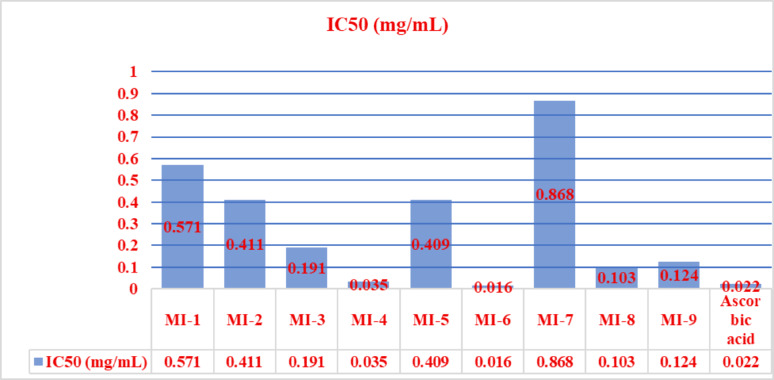
Comparison of the antioxidant results expressed as IC_50_ in mg/mL of the tested samples relative to the antioxidant standard from **MI-1** to **MI-9**

The antioxidant activity of the carbohydrazide derivatives **MI-1**–**MI-9** was assessed using the DPPH• radical-scavenging assay, and the results are summarized in Table 2 and shown in Figs. 5, 6 All compounds exhibited varying degrees of free-radical-scavenging capacity, as indicated by their % scavenging values and corresponding IC_50_ parameters [25].

**Table 2:** The antioxidant results by DPPH assay

Samples	% Remaining DPPH	% Scavenging Activity	IC_50_ (mg/mL)	Relative Activity (% of Ascorbic Acid)
**MI-1**	25.17	74.83	0.571	88.27
**MI-2**	22.38	77.62	0.411	91.68
**MI-3**	12.59	87.41	0.016	103.17
**MI-4**	19.86	80.14	0.035	94.59
**MI-5**	66.41	33.59	0.409	39.64
**MI-6**	38	62.03	0.191	73.21
**MI-7**	48.1	51.89	0.868	61.24
**MI-8**	54.55	45.45	0.103	53.64
**MI-9**	20.56	79.44	0.124	93.75
Ascorbic acid	15.27	84.73	0.022	100

The relationship between the structure and activity in the DPPH test for these compounds is influenced by groups that can donate electrons and those that can stabilize radicals. The ability to give away hydrogen atoms and stabilize radicals through resonance improves the antioxidant activities of **MI-6**, **MI-5**, and **MI-4** when they have -SH and -C = S groups. Electron delocalization, brought about by heterocyclic systems that include oxadiazole, triazole, pyrazole, and benzothiazole, enhances the radical quenching efficiency. Compounds **MI-8** and **MI-9** are able to successfully scavenge radicals thanks to their benzothiazole and indole groups, which allow electron transfer to DPPH radicals through conjugated π-systems. Hydrazine (-NH-NH_2_) and hydrazone (-C = NNH-) are radical stabilizers that are included in **MI-4** and **MI-3**, respectively. They do this by creating hydrogen bonding possibilities and electron transfer capabilities. Pyrazole derivatives **MI-1** and **MI-2** lack strong electron donor groups like -SH or **-C = S**, which is why they are not as effective as antioxidants compared to other substances. Molecular sulfur-containing functionalities, extensive conjugation networks, and heterocyclic systems that allow electron delocalization and radical stabilization are the primary determinants of antioxidant strength in these compounds.

Among the tested compounds, **MI-3** showed the most potent antioxidant activity (IC_50_ = 0.016 mg/mL), surpassing ascorbic acid (IC_50_ = 0.022 mg/mL). Correspondingly, MI-3 exhibited the highest relative activity (103.17%), confirming its superior radical-scavenging efficiency. **MI-4** followed with a significantly low IC_50_ value (0.035 mg/mL) and high relative activity (94.59%), indicating strong antioxidant potential comparable to the standard. Compounds with higher IC_50_ values, such as **MI-5** and **MI-7**, displayed markedly lower relative activity, reflecting weaker scavenging power. Overall, the results clearly demonstrate that the antioxidant efficacy of the synthesized compounds correlates well with their IC_50_ values, validating IC_50_ as a reliable indicator of free radical-scavenging capacity. The excellent activity of **MI-3** and MI-4 may be attributed to structural features such as electron-donating substituents and conjugation, which facilitate hydrogen atom transfer and electron donation to stabilize free radicals.

## Molecular docking study

**Fig. 7:**
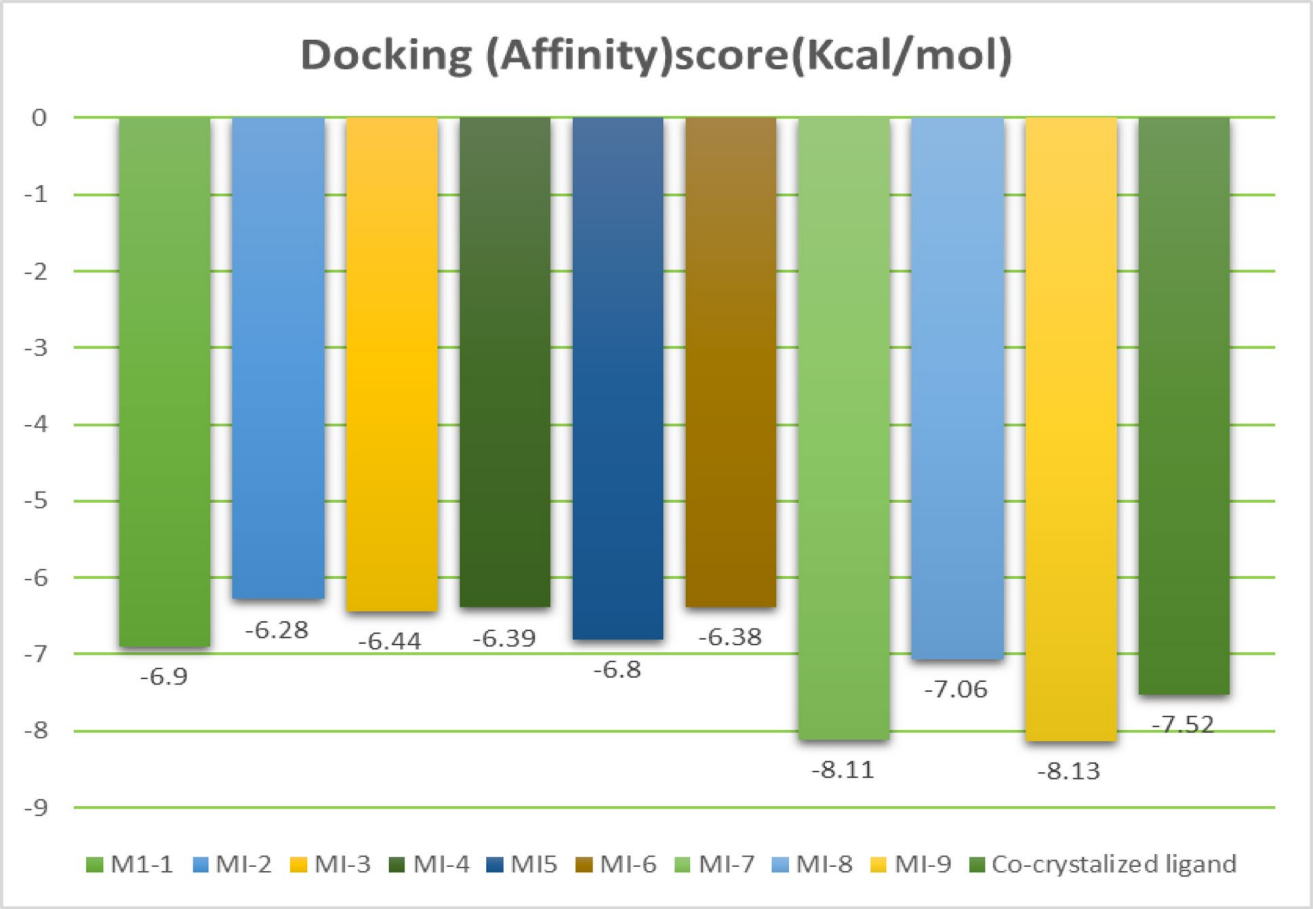
Docking (Affinity) score of compounds **MI-1-9**

**Fig. 8:**
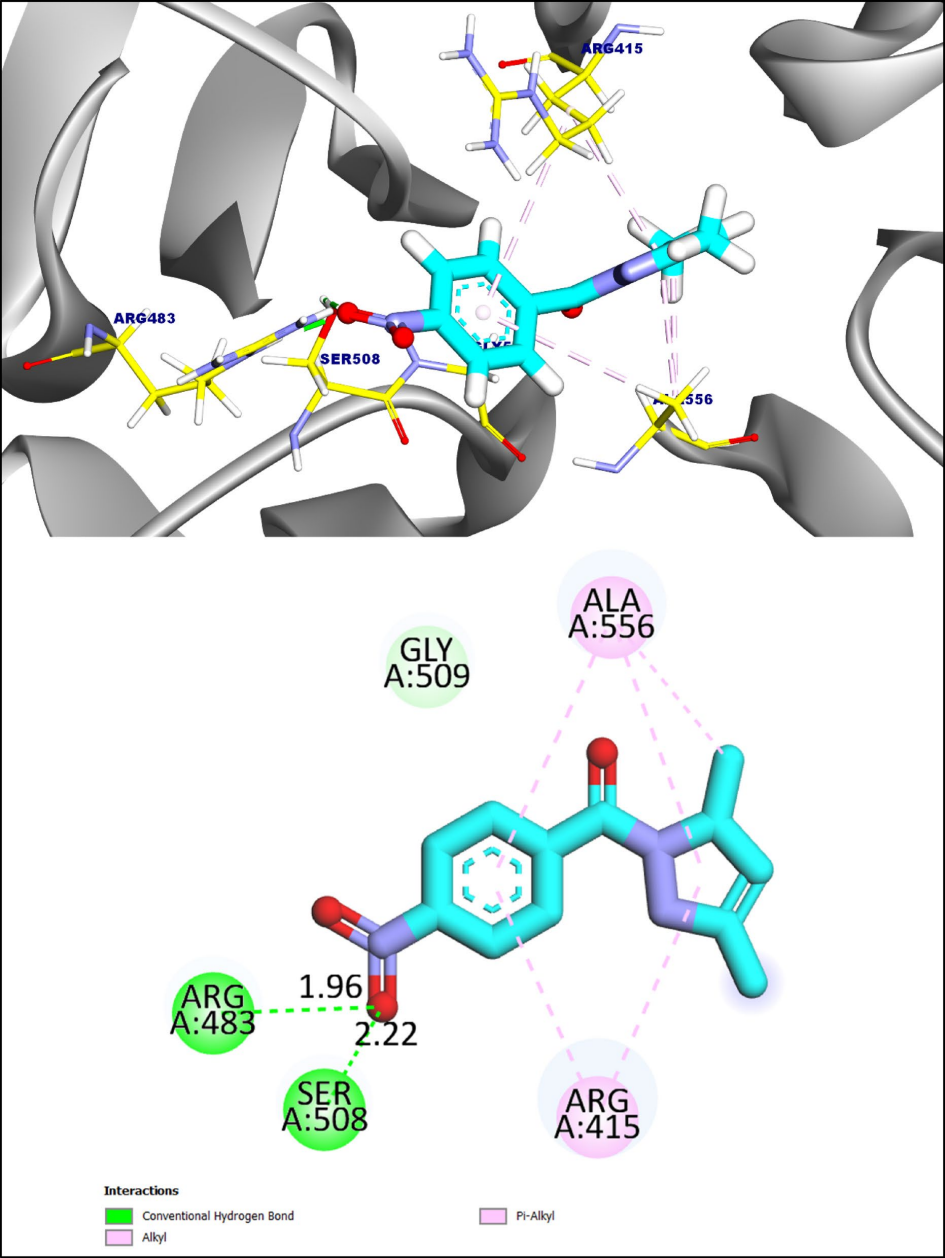
3D and 2D figures of the proposed binding mode of **compound MI-1** against **Keap1-nrf2 complex**

**Fig. 9:**
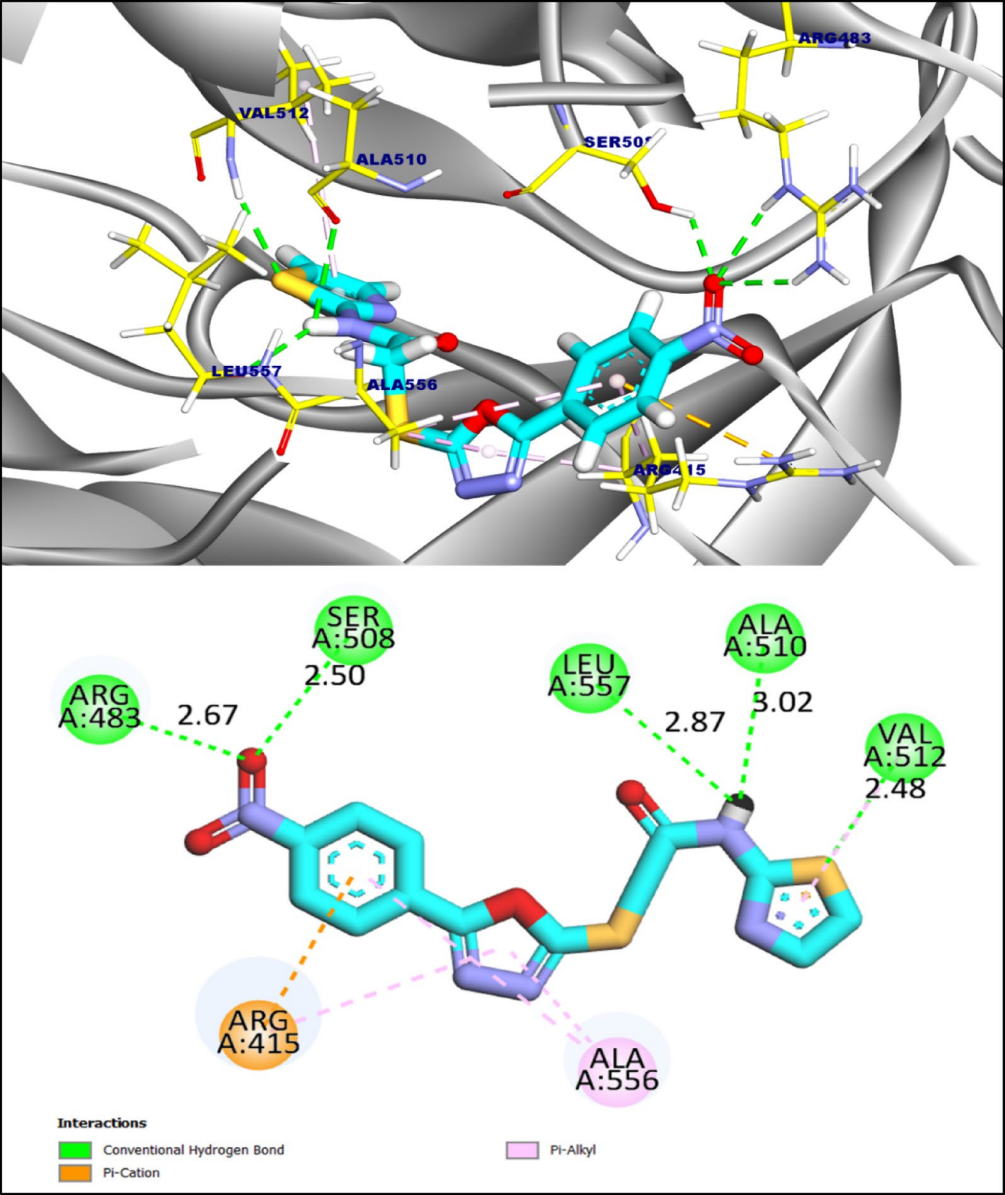
3D and 2D figures of the proposed binding mode of compound **MI-5** against Keap1-nrf2 complex

**Fig. 10:**
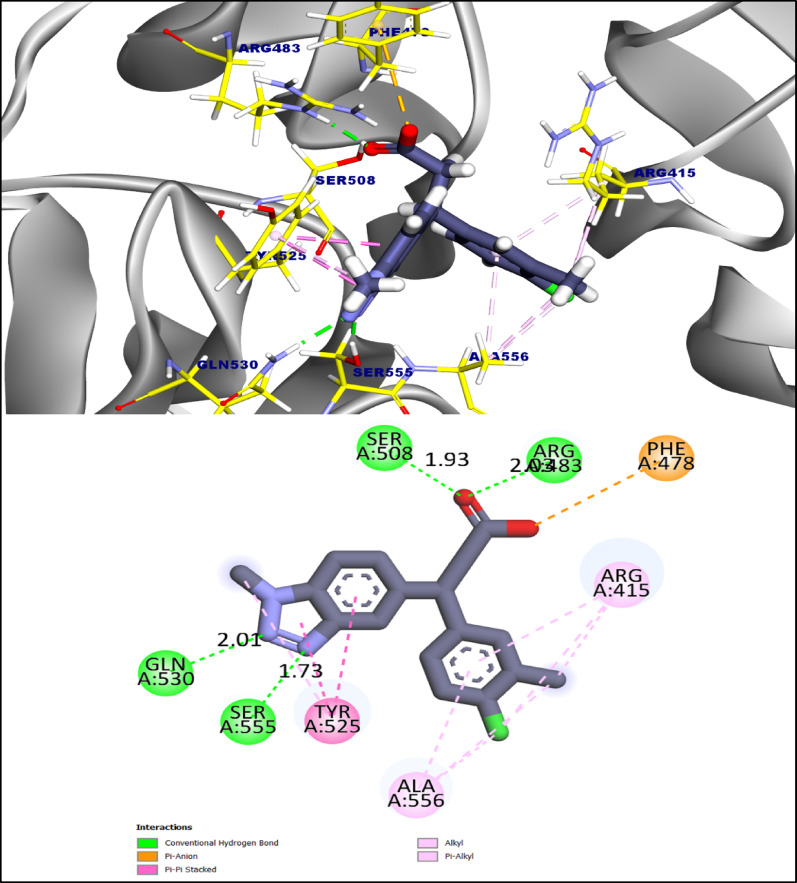
3D and 2D figures of the proposed binding mode of compound **MI-9** against **Keap1-nrf2 complex**

**Fig. 11:**
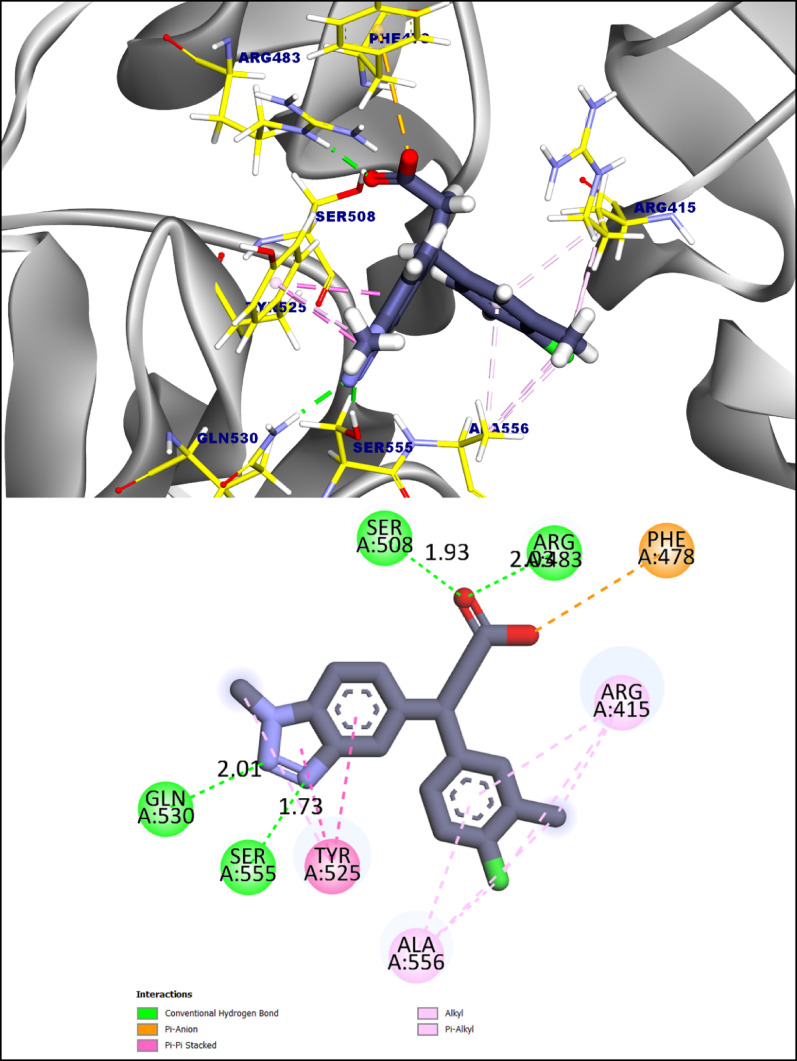
showed 3D and 2D figures of the proposed binding mode of the co-crystalized ligand complexed with **Keap1-nrf2** complex

The tested compounds were docked against the **Keap1-nrf2** complex to evaluate their potential binding affinity as potential antioxidant agents. The results of the top docked compounds are illustrated in Table 3; Fig. 7 [32]. To elucidate the binding interactions of the synthesized indole–hydrazone derivatives with the **Keap1–Nrf2** interface, all ligands were docked into the Kelch domain of Keap1. The docking results (Table 3; Figs. 8, 9, 10 and 11) revealed that compounds **MI-7** and **MI-9** exhibited the most favorable binding energies (-8.11 and − 8.13 kcal·mol^− 1^, respectively), outperforming the co-crystallized ligand (-7.52 kcal·mol^− 1^).

**Table 3:** Molecular Docking analysis of the tested compounds against Keap1-nrf2 complex

Tested compounds	Compounds	RMSD value (Å)	Docking (Affinity) score (kcal/mol)
Keap1-nrf2 complex	**MI-1**	1.43	-6.90
	**MI-2**	1.81	-6.28
	**MI-3**	1.36	-6.44
	**MI-4**	1.53	-6.39
	**MI-5**	0.79	-6.80
	**MI-6**	0.96	-6.38
	**MI-7**	1.55	-8.11
	**MI-8**	1.12	-7.06
	**MI-9**	1.39	-8.13
	**Co-crystalized ligand**	1.07	-7.52

### Molecular docking of target compounds against Keap1-nrf2 complex (nrf2 activation)

Compound **MI-1** demonstrated a reasonable binding affinity (-6.90 kcal·mol⁻¹). **MI-**1 adopted a stable pose within the Keap1 binding pocket, forming two hydrogen bonds with Arg483 and Ser508 at distances of 1.96 and 2.22 Å, respectively, along with five π-alkyl interactions involving Ala556 and Arg415, which contributed to hydrophobic stabilization. (Fig. 8).

Compound **MI-5** showed a **favorable** predicted affinity toward the Keap1 Kelch cavity (docking score − 6.80 kcal·mol⁻¹). In the top docking pose **MI-5** adopts an oriented conformation within the groove and is stabilized by multiple polar and hydrophobic contacts. Five hydrogen bonds were identified with Arg483, Ser508, Leu557, Ala510 and Val512 (ligand atom → protein acceptor/donor distances ≈ 2.48–3.02 Å), indicating engagement of both side-chain and backbone atoms. These polar interactions are complemented by a π–cation contact with Arg415 and π–alkyl contacts with Ala556 and Val512, which collectively contribute to aromatic stacking and hydrophobic packing inside the binding site. Taken together, the docking results suggest **MI-5** is a viable Keap1 binder; however, given the intrinsic uncertainties of docking scores, biochemical or cell-based assays are required to confirm any **Nrf2-**modulating activity. (Fig. 9).

**MI-9**, the best-scoring compound (− 8.13 kcal·mol⁻¹), displayed extensive hydrogen-bonding capability, forming four hydrogen bonds with Ser508, Arg483 (two interactions), and Gly364 at distances of 2.04–3.00 Å. Furthermore, **MI-9** engaged in eleven hydrophobic and π-mediated interactions, including π–π stacking with Tyr572, Phe577, and Tyr334, π-anion and π-cation contacts with Arg415, and π-alkyl interactions with Ala556 and Phe478. These interactions suggest deeper insertion and enhanced aromatic stabilization within the cavity relative to other ligands. (Fig. 10).

For comparison, the co-crystallized ligand bound with a predicted affinity of -7.52 kcal·mol⁻¹, forming four hydrogen bonds with Ser508, Arg483, Gln530, and Ser555 (1.73–2.03 Å) and ten hydrophobic/π interactions with Phe478, Arg415, Ala556, and Tyr525. Notably, MI-7 and MI-9 reproduced key interactions observed for the reference ligand, particularly with Arg483 and Ser508, while providing additional hydrogen-bonding sites and enhanced π-stacking networks, rationalizing their superior predicted binding affinities and potential as Keap1–Nrf2 modulators (Fig. 11) [27].

To evaluate the potential of the synthesized carbohydrazide derivatives to activate the **Nrf2** pathway, molecular docking was performed targeting the Kelch domain of Keap1, the principal binding interface responsible for **Nrf2** recognition. Docking scores and binding interactions were compared across the series and benchmarked against the co-crystallized reference ligand. Results are summarized in Table 3. Among the tested compounds, **MI-7** (− 8.11 kcal/mol) and **MI-9** (− 8.13 kcal/mol) demonstrated the highest binding affinities, exceeding the co-crystallized ligand (− 7.52 kcal/mol), indicating their strong potential to competitively disrupt Keap1–**Nrf2** interactions. Compounds **MI-1**, **MI-5**, and **MI-8** also exhibited moderate activity, with docking scores in the − 6.80 to − 7.06 kcal/mol range, while **MI-2-MI-4** and **MI-6** displayed comparatively lower affinity. Consistent with reported Keap1 inhibitor motifs in literature, the most active compounds reproduced key interactions with critical residues such as **Arg483 and Ser508**, which are essential for Nrf2 binding, while also forming additional stabilizing contacts within the Tyr334/Tyr572 aromatic clamp region. Similar interaction patterns have been highlighted in previously reported Keap1 inhibitors bearing heteroaromatic scaffolds and hydrogen-bond-rich pharmacophores.

###  Relationship between molecular Docking scores and antioxidant potency

The molecular docking study indicated that MI-9 and MI-7 rank highest for predicted affinity toward the Keap1–Nrf2 binding site (− 8.13 and − 8.11 kcal.mol⁻¹, respectively), slightly exceeding the co-crystallized ligand (− 7.52 kcal.mol⁻¹). Visual inspection of the top docking poses reveals that these compounds place the oxadiazole-thioacetamide motifs into key sub-pockets and form multiple polar contacts and hydrogen bonds, which likely contribute to the favorable scoring [33]. Importantly, these in silico binding rankings do not directly translate into superior DPPH radical scavenging: the best DPPH performers were **MI-3** and **MI-4** (IC_50_ = 0.016 and 0.035 mg·mL^− 1^, respectively), whereas **MI-7** and **MI-9** showed weaker DPPH activity. This divergence is not unexpected because the two assays probe different properties: DPPH measures direct radical quenching (primarily via H-atom transfer or single-electron transfer under the test conditions), which depends on accessible hydrogen donors, bond dissociation energies and local electron density; docking scores estimate a compound’s propensity to be retained in a protein pocket via noncovalent interactions (hydrogen bonds, π-stacking, electrostatics). Thus, **MI-3** and **MI-4**—bearing isoindolinone and thioamide functionalities—present readily accessible H-donor/electron-rich sites that favor rapid chemical reduction of DPPH, whereas MI-7 and **MI-9** possess scaffolds and substituents that enhance protein pocket complementarity and polar interactions but not necessarily intrinsic redox reactivity. We also caution that docking scores have intrinsic uncertainties (often ≈ 1 kcal·mol^− 1^) and that very small score differences (e.g., the 0.02 kcal·mol^− 1^ difference between **MI-9** and **MI-7**) should not be overinterpreted. To strengthen the functional relevance of the docking results, future work could include rescoring, molecular dynamics of the best poses, and cell-based assays for **Nrf2** activation to complement the in vitro DPPH data.

## Conclusion

In this study, a novel series of hydrazide–hydrazone derivatives incorporating triazole and oxadiazole scaffolds (**MI-1 to MI-9**) was successfully synthesized and thoroughly characterized using various spectroscopic methods. The in vitro antioxidant evaluation using the DPPH assay revealed that several derivatives, particularly **MI-3** and **MI-4**, exhibited potent radical scavenging activity, in some cases surpassing ascorbic acid. Density functional theory (DFT) calculations provided insight into the electronic properties and reactivity of the synthesized compounds, where lower HOMO-LUMO energy gaps, greater softness, and extended orbital delocalization correlated strongly with enhanced antioxidant performance. Frontier molecular orbital (FMO) analysis highlighted that compounds with highly delocalized HOMO densities, such as **MI-3** and **MI-5**, are more efficient electron donors, contributing to their superior radical-quenching ability. Molecular docking studies against the Keap1–Nrf2 complex further demonstrated favorable binding interactions for key derivatives (**MI-7**,** MI-8**, and **MI-9**), suggesting their potential to modulate the Nrf2 antioxidant pathway. Overall, the combined experimental and computational findings underscore the promising antioxidant potential of these novel hydrazide–hydrazone derivatives and provide a robust structure–activity framework for the future development of multifunctional therapeutic agents targeting oxidative stress, related disorders.

## Supplementary Information

Below is the link to the electronic supplementary material.

Supplementary Material 1

## Data Availability

The datasets used and/or analysed during the current study are available from the corresponding author on reasonable request.

## References

[1] Aalami-Harandi R, Karamali M, Asemi Z, RETRACTED ARTICLE. The favorable effects of Garlic intake on metabolic profles, hs-CRP, biomarkers of oxidative stress and pregnancy outcomes in pregnant women at risk for pre-eclampsia: Randomized, double-blind, placebo-controlled trial. J Maternal-Fetal Neonatal Med. 2015;28(17):2020–7. 10.3109/14767058.2014.977248 25316559

[2] Farzaliyeva A, Şenol H, Taslimi P, Çakır F, Farzaliyev V, Sadeghian N, Gulcin I. Synthesis and biological studies of acetophenone-based novel chalcone, semicarbazone, thiosemicarbazone and Indolone derivatives: Structure-Activity relationship, molecular docking, molecular dynamics, and kinetic studies. J Mol Struct. 2025;1321:140197.

[3] Amine Khodja I, Boulebd H. Synthesis, biological evaluation, theoretical investigations, Docking study and ADME parameters of some 1, 4-bisphenylhydrazone derivatives as potent antioxidant agents and acetylcholinesterase inhibitors. Mol Divers. 2021;25(1):279–90. 32146656 10.1007/s11030-020-10064-8

[4] Gulcin İ, Alwasel SH. Metal ions, metal chelators and metal chelating assay as antioxidant method. Processes. 2022;10(1):132.

[5] Kolak U, HACIBEKİROĞLU I, Öztürk M, Özgökçe F, Topcu G, Ulubelen A. (2009). Antioxidant and anticholinesterase constituents of Salvia poculata. *Turk. J. Chem*. 2009; *33*(6), 813–823.

[6] Mateev E, Muhammed MT, Irfan A, Sharma S, Georgieva M, Zlatkov A. Hydrazide-hydrazones as novel antioxidants-in vitro, molecular Docking and DFT studies. Pharmacia. 2024;71:1–8.

[7] Vinci G, D’Ascenzo F, Maddaloni L, Prencipe SA, Tiradritti M. The influence of green and black tea infusion parameters on total polyphenol content and antioxidant activity by ABTS and DPPH assays. Beverages. 2022;8(2):18.

[8] El-Zawawy, Reham O, et al. Pyrazole-pyridine-carbohydrazone hybrids as promising antioxidants: synthesis, characterization, DFT, ADME, and molecular Docking studies on cyclooxygenase-2. J Mol Struct. 2025;1337:142201.

[9] Ugwu JC, et al. Investigating the antioxidant potential and mechanism of a Hydrazide bioactive component of garlic: insights from density functional theory calculations, drug-likeness and molecular Docking studies. Appl Biochem Biotechnol. 2025;1972:847–72. 10.1007/s12010-024-05051-w 39292337

[10] Boulebd H, et al. Synthesis and radical scavenging activity of new phenolic hydrazone/hydrazide derivatives: experimental and theoretical studies. J Mol Struct. 2022;1249:131546.

[11] Abu-Hashem AA. Synthesis and antimicrobial activity of new 1, 2, 4‐triazole, 1, 3, 4‐oxadiazole, 1, 3, 4‐thiadiazole, thiopyrane, thiazolidinone, and Azepine derivatives. J Heterocycl Chem. 2021;58(1):74.

[12] Lemilemu F, Bitew M, Demissie TB, Eswaramoorthy R, Endale M. Synthesis, antibacterial and antioxidant activities of Thiazole-based schiff base derivatives: a combined experimental and computational study. BMC Chem. 2021;15(1):67. 34949213 10.1186/s13065-021-00791-w PMC8697436

[13] Mahmood A, Khan SUD, Rana UA, Tahir MH. Red shifting of absorption maxima of phenothiazine based dyes by incorporating electron-deficient thiadiazole derivatives as π-spacer. Arab J Chem. 2019;12(7):1447–53.

[14] Mahmood A. Photovoltaic and charge transport behavior of Diketopyrrolopyrrole based compounds with A–D–A–D–A skeleton. J Cluster Sci. 2019;30:1123–30.

[15] Khan UK, Mehboob MY, Hussain R, Afzal Z, Khalid M, Adnan M. Designing spirobifullerene core based three-dimensional cross shape acceptor materials with promising photovoltaic properties for high‐efficiency organic solar cells. Int J Quantum Chem 120(22).

[16] Khan MU, Mehboob MY, Hussain R, Fatima R, Tahir SM, Khalid M, Braga AAC. Molecular designing of high-performance 3D star‐shaped electron acceptors containing a Truxene core for nonfullerene organic solar cells. J Phys Org Chem 2021; 34(1), e4119.

[17] Frisch A. gaussian 09 W Reference. Wallingford, Usa.2009; 25: 470.

[18] Abu-Awwad F, Politzer P. Variation of parameters in Becke‐3 hybrid exchange‐correlation functional. J Comput Chem. 2000;21(3):227–38.

[19] Check CF, Gilbert TM. Progressive systematic underestimation of reaction energies by the B3LYP model as the number of C – C bonds increases: why organic chemists should use multiple DFT models for calculations involving polycarbon hydrocarbons. J Org Chem. 2005;70(24):9828–34. 16292812 10.1021/jo051545k

[20] Krishnan RBJS, Binkley JS, Seeger R, Pople JA. Self-consistent molecular orbital methods. XX. A basis set for correlated wave functions. J Chem Phys. 1980;72(1):650–4.

[21] McLean AD, Chandler GS. Contracted Gaussian basis sets for molecular calculations. I. Second row atoms, Z = 11–18. J Chem Phys. 1980;72(10):5639–48.

[22] Clark T, Chandrasekhar J, Spitznagel GW, Schleyer PVR. Efficient diffuse function-augmented basis sets for anion calculations. III. The 3‐21 + G basis set for first‐row elements, Li–F. J Comput Chem. 1983;4(3):294–301.

[23] Tirado-Rives J, Jorgensen WL. Performance of B3LYP density functional methods for a large set of organic molecules. J Chem Theory Comput. 2008;4(2):297–306. 26620661 10.1021/ct700248k

[24] Miar M, Shiroudi A, Pourshamsian K, Oliaey AR, Hatamjafari F. Theoretical investigations on the HOMO–LUMO gap and global reactivity descriptor studies, natural bond orbital, and nucleus-independent chemical shifts analyses of 3-phenylbenzo [d] thiazole-2 (3 H)-imine and its para-substituted derivatives: solvent and substituent effects. J Chem Res. 2021;45(1–2):147–58.

[25] Gulcin İ, Alwasel SH. DPPH radical scavenging assay. Processes. 2023;11(8):2248.

[26] Olszowy-Tomczyk M. How to express the antioxidant properties of substances properly? Chem Pap. 2021;75(12):6157–67.

[27] Bakheit AH, Wani TA, Al-Majed AA, Alkahtani HM, Alanazi MM, Alqahtani FR, Zargar S. Theoretical study of the antioxidant mechanism and structure-activity relationships of 1, 3, 4-oxadiazol-2-ylthieno [2, 3-d] pyrimidin-4-amine derivatives: a computational approach. Front Chem. 2024;12:1443718. 39139921 10.3389/fchem.2024.1443718 PMC11319267

[28] Ismael M, Abdel-Mawgoud AMM, Rabia MK, Abdou A. Design and synthesis of three Fe (III) mixed-ligand complexes: exploration of their biological and phenoxazinone synthase-like activities. Inorg Chim Acta. 2020;505:119443.

[29] Alisi IO, Uzairu A, Abechi SE. In Silico design of hydrazone antioxidants and analysis of their free Radical-Scavenging mechanism by thermodynamic studies. Beni-Suef Univ J Basic Appl Sci. 2019; 8, Article 11.

[30] Tzankova D, Kuteva H, Mateev E, Stefanova D, Dzhemadan A, Yordanov Y, Georgieva M, Synthesis. DFT study, and in vitro evaluation of antioxidant properties and cytotoxic and cytoprotective effects of new hydrazones on SH-SY5Y neuroblastoma cell lines. Pharmaceuticals. 2023;16(9):1198. 37765006 10.3390/ph16091198 PMC10537553

[31] dos Santos MAB, de Oliveira LFS, de Figueiredo AF, dos, Santos Gil F, de Souza Farias M, Bitencourt HR, Ciríaco-Pinheiro J. Molecular electrostatic potential and chemometric Techniques as Tools to Design Bioactive Compounds. In *Cheminformatics and its Applications*. 2019.

[32] Huang Z, Peng Z, Huang D, Zhou Z. Virtual screening of Kelch-like ECH-Associated protein 1-Nuclear factor erythroid 2-Related factor 2 (Keap1-Nrf2) inhibitors and in vitro validation. Molecules. 2025;30(8):1815. 40333848 10.3390/molecules30081815 PMC12029559

[33] Huang Li M, Jie W, Wang F, Zhong M, Chen Y, Q., Lu B. Discovery of Keap1 – Nrf2 small – molecule inhibitors from phytochemicals based on molecular Docking. Food Chem Toxicol. 2029;133:110758. 10.1016/j.fct.2019.110758 PMC7116978 31412289

