# Supplementary material for: Design, synthesis, structural characterization, and antioxidant potential of novel triazole- and oxadiazole-based hydrazide-hydrazone derivatives: spectroscopic, DFT, and molecular docking studies
Source: BMC Chem. 2025 Dec 24;20(1):19. doi: 10.1186/s13065-025-01698-6 (PMC12849251; doi:10.1186/s13065-025-01698-6)
Supplement: Supplementary file 1 — Supplementary Material 1 [file 13065_2025_1698_MOESM1_ESM.docx]

**Design, Synthesis, Structural Characterization, and Antioxidant Potential of Novel Triazole- and Oxadiazole-Based Hydrazide-Hydrazone Derivatives: Spectroscopic, DFT, and Molecular Docking Studies**

Mariam A. Abdo^a1^, Safa A. Badawy^a*1^, Ahmed A. Fadda^a,1^, Mohamed R. Elmorsy^a*1^,

*^a^Department of Chemistry, Faculty of Science, Mansoura University, El-Gomhoria Street, 35516 Mansoura, Egypt.*

*^*^ Corresponding author: E-mail:* [m.r.elmorsy@gmail.com](mailto:m.r.elmorsy@gmail.com), [safabadawy140@gmail.com](mailto:safabadawy140@gmail.com).

***1. Materials and methods:***

The chemicals and solvents necessary for the chemical reactions and synthetic procedures were acquired from Sigma-Aldrich, TCI America, and Alfa Aesar, and utilized exactly as supplied. The measured melting points (uncorrected) are just in degrees Celsius, employing a Gallenkamp electric melting point instrument. A Thermo Scientific Nicolet iS10 FTIR spectrometer was used for identifying the IR spectra (KBr). Nuclear magnetic resonance (NMR) spectra were obtained on a Bruker NMR spectrometer using DMSO-*d*_6_ as a solvent at 400 and 500 MHz (^1^H NMR) and 100 MHz (^13^C NMR) with an internal standard (TMS), and chemical shifts are given as δ/ppm. The UV-Visible spectra were measured by using the high-performance double beam spectrophotometer (T80 series). The mass studies were performed using a Thermo DSQ II spectrometer. The elemental analysis data was collected using the Perkin Elmer 2400 analyzer. Finally, all instruments and DSSC fabrications are thoroughly discussed in the attached information file

**2. Molecular Docking Analysis**

Molecular docking was pocessed to evaluate the potential affinity of the tested compounds as potential antioxidant agents. the tested compounds were docked against **Keap1-nrf2 complex** , which obtained from Protein Data Bank (PDB code: 6qme)[1]. At first, water molecules was neglected and unnessesary molecules were removed from the protein complex. then, any complex disorders and unfilled valence atoms were corrected. The protein structure energy was minimized and saved as PDBQT files .The 2D structure of each compound was drawn using Chem-Bio Draw Ultra16.0, saved as an SDF file, and then converted to a 3D structure. Protonation and energy minimization were carried out and saved as PDBQT files , The docking porcsses conducted using Autodock Vina 1.5.7 software . the docking was conducted by rigid technique, in this method the receptor was held rigid while the ligands were allowed to be flexible[2]. Additionally, each molecule was allowed to generate twenty different poses. The docking scores (affinity energy) of the best-fitted poses with the target protein were recorded, and 3D and 2D figures were generated using Discovery Studio 2024 visualizer[3].

1. Heightman, T.D., et al., *Structure–activity and structure–conformation relationships of aryl propionic acid inhibitors of the Kelch-like ECH-associated protein 1/nuclear factor erythroid 2-related factor 2 (KEAP1/NRF2) protein–protein interaction.* Journal of medicinal chemistry, 2019. **62**(9): p. 4683-4702.

2. Saleh, A.M., et al., *Design, synthesis, in silico studies, and biological evaluation of novel pyrimidine-5-carbonitrile derivatives as potential anti-proliferative agents, VEGFR-2 inhibitors and apoptotic inducers.* RSC advances, 2023. **13**(32): p. 22122-22147.

3. El-Demerdash, A.S., et al., *Essential oils as capsule disruptors: enhancing antibiotic efficacy against multidrug-resistant Klebsiella pneumoniae.* Frontiers in Microbiology, 2024. **15**: p. 1467460.

**3. Figures**


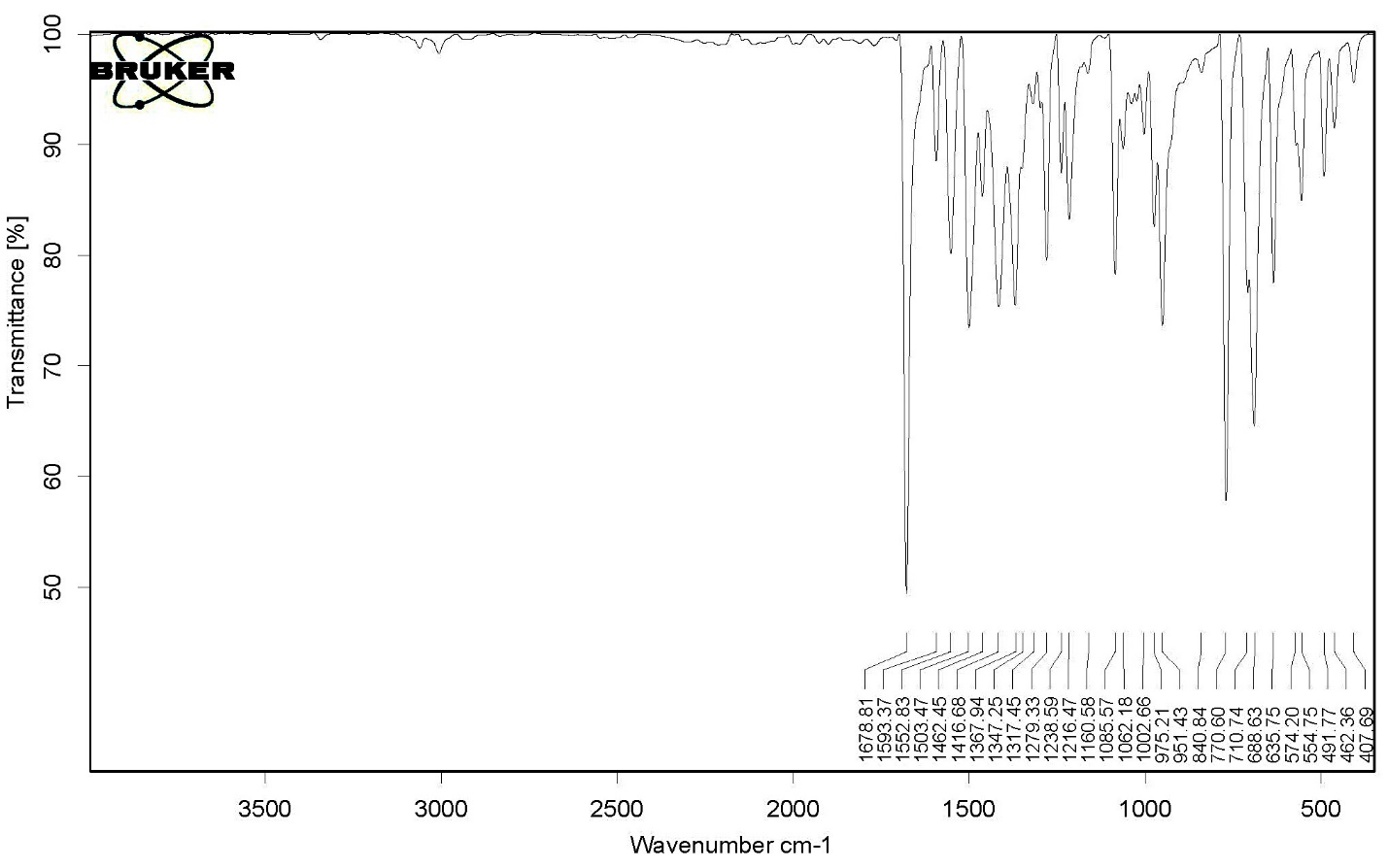


**Fig (S1): IR spectrum of compound MI-1.**


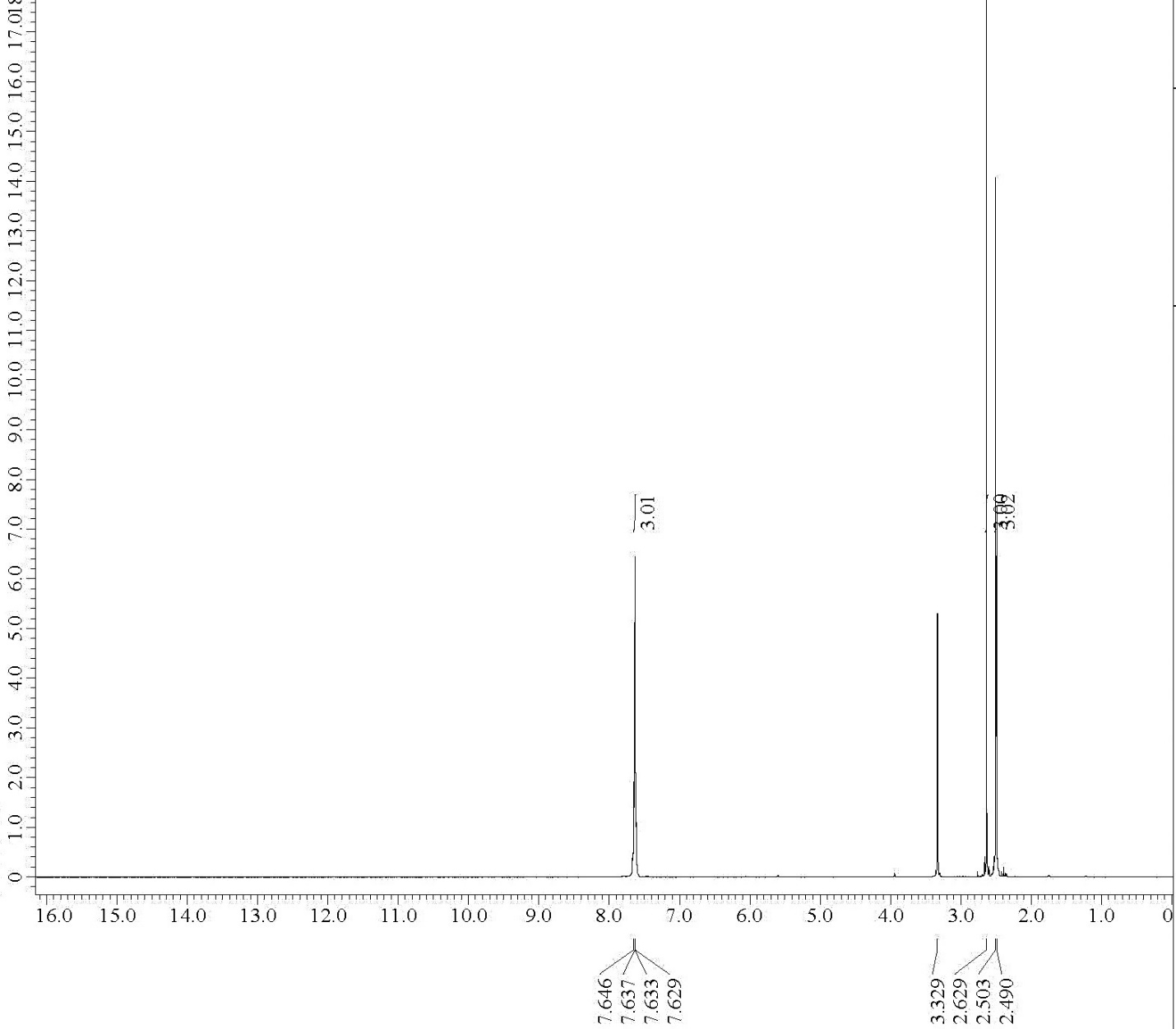


**Fig (S2): ^1^H NMR spectrum of compound MI-1.**


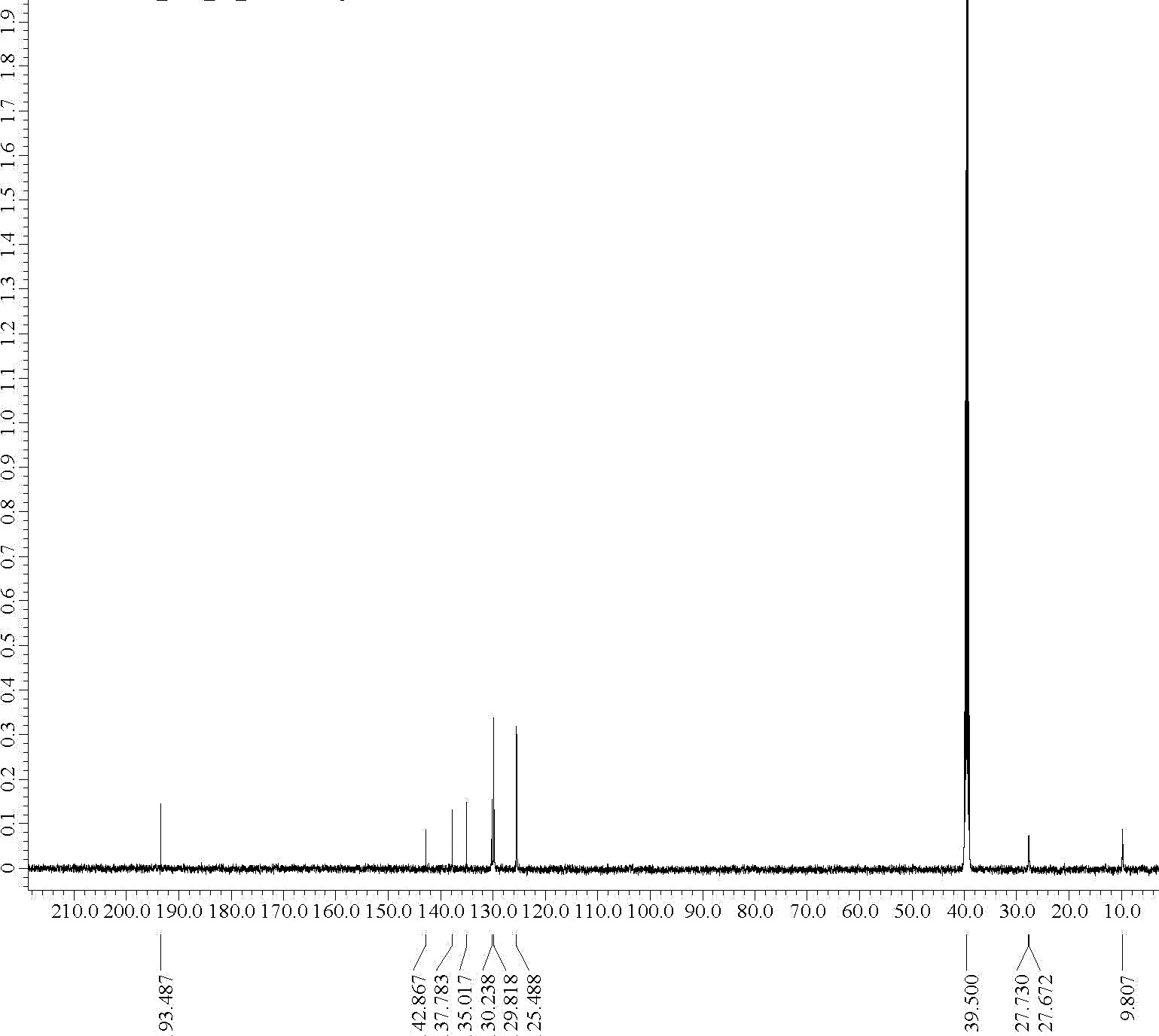


**Fig (S3): ^13^C NMR spectrum of compound MI-1.**

** Fig (S4): Mass spectrum of compound MI-1.**


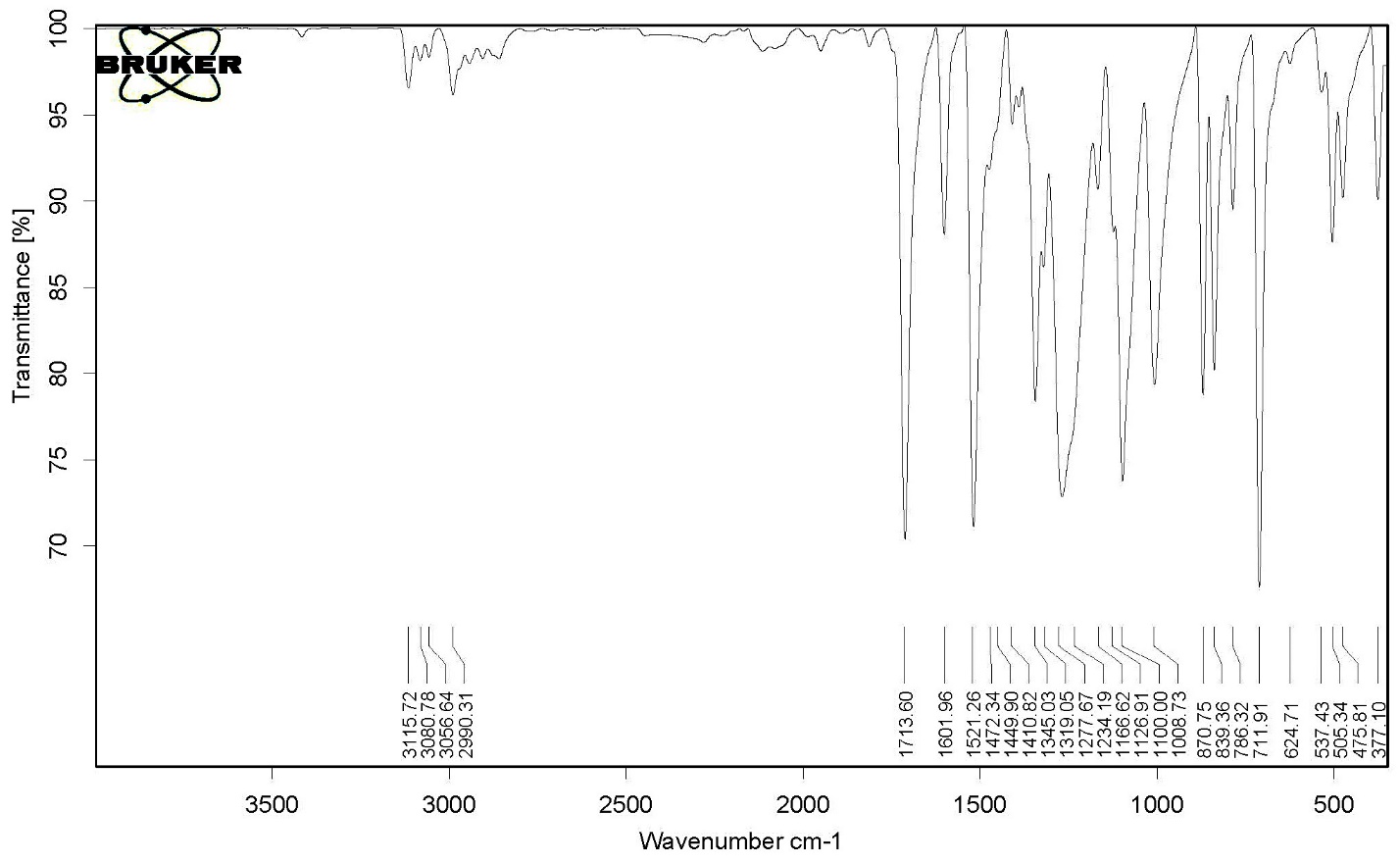
 **Fig (S5): IR spectrum of compound MI-2.**


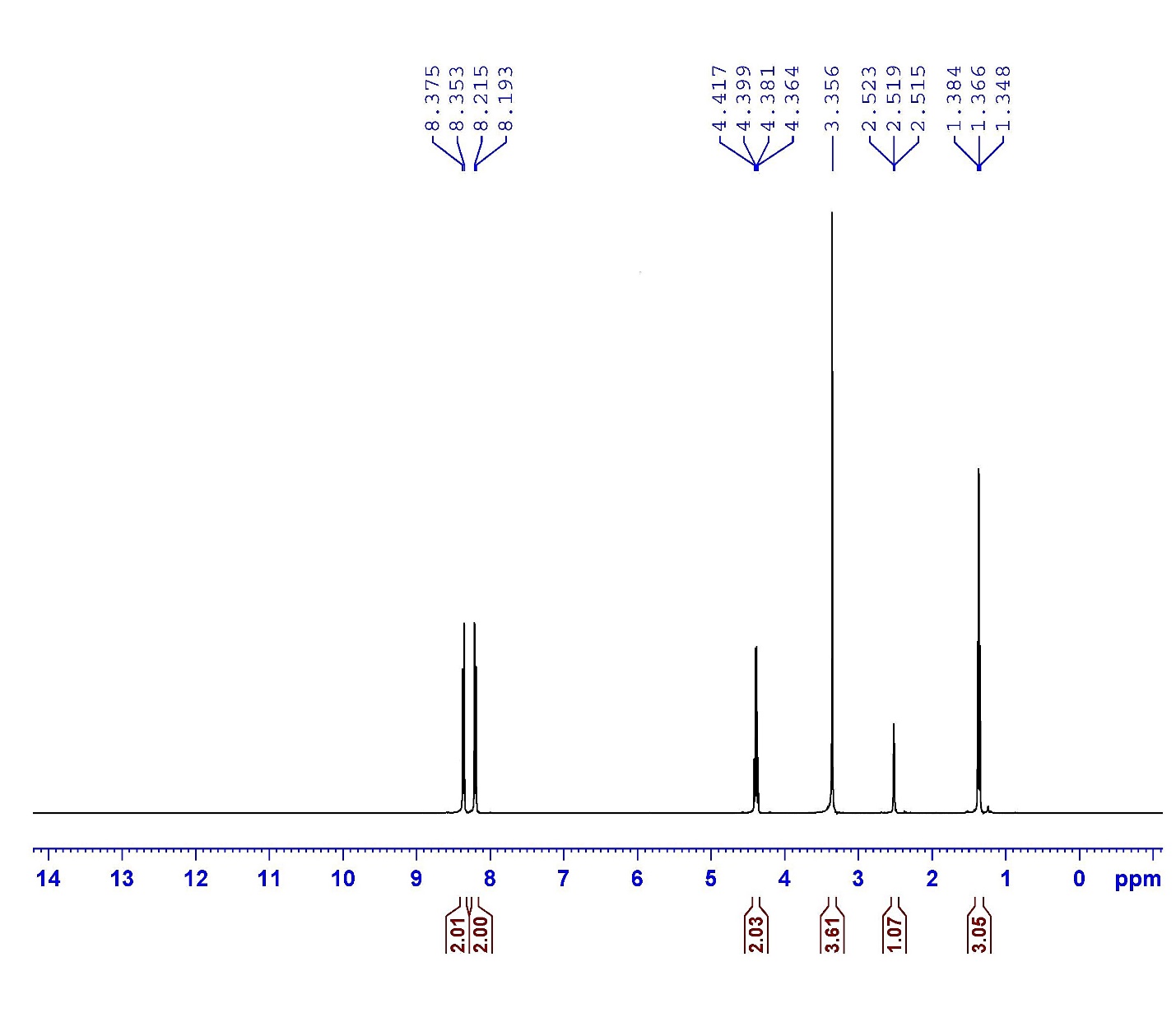
 **Fig (S6): ^1^H NMR spectrum of compound MI-2.**


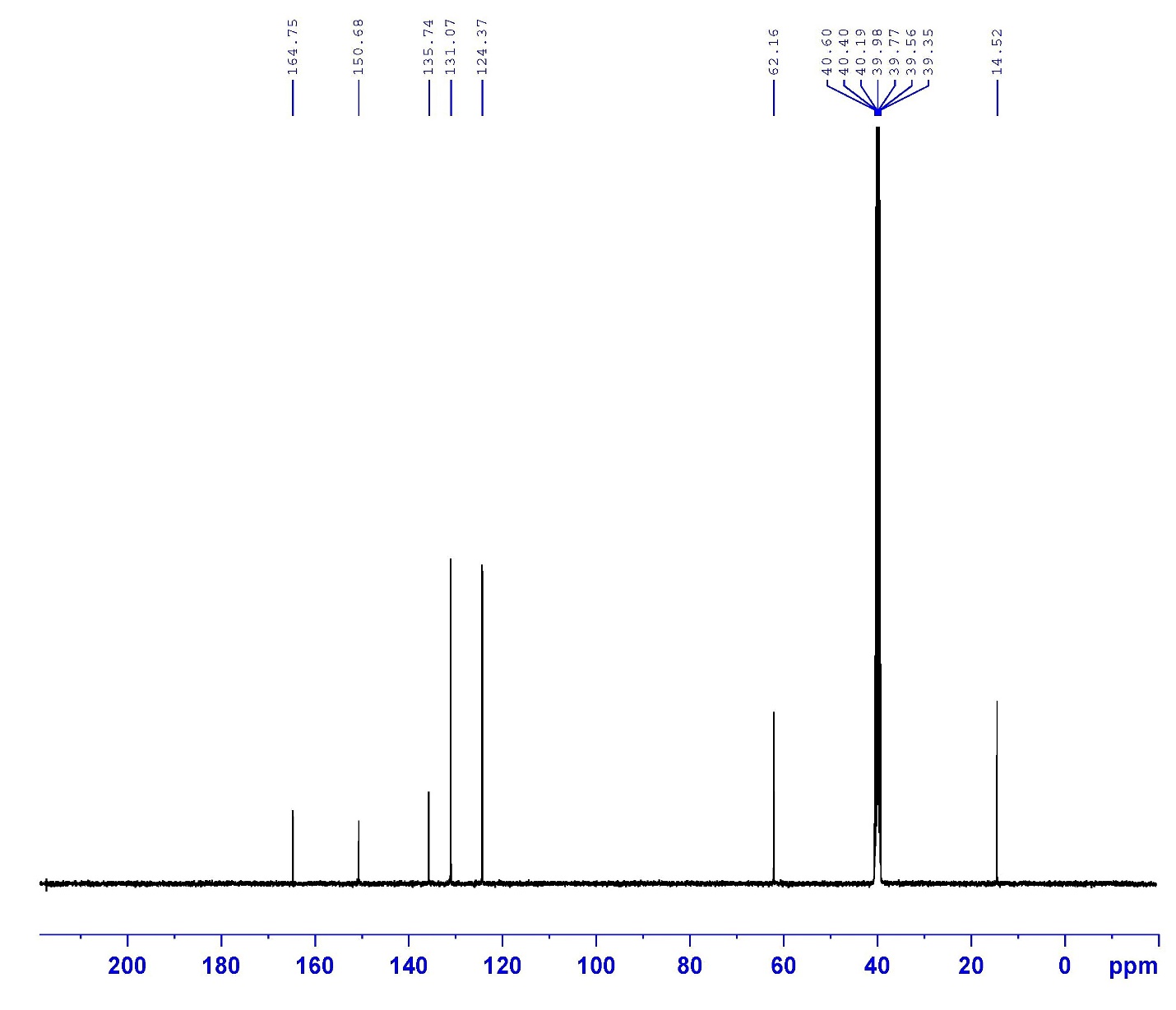
 **Fig (S7): ^13^C NMR spectrum of compound MI-2.**

** Fig (S8): Mass spectrum of compound MI-2.**


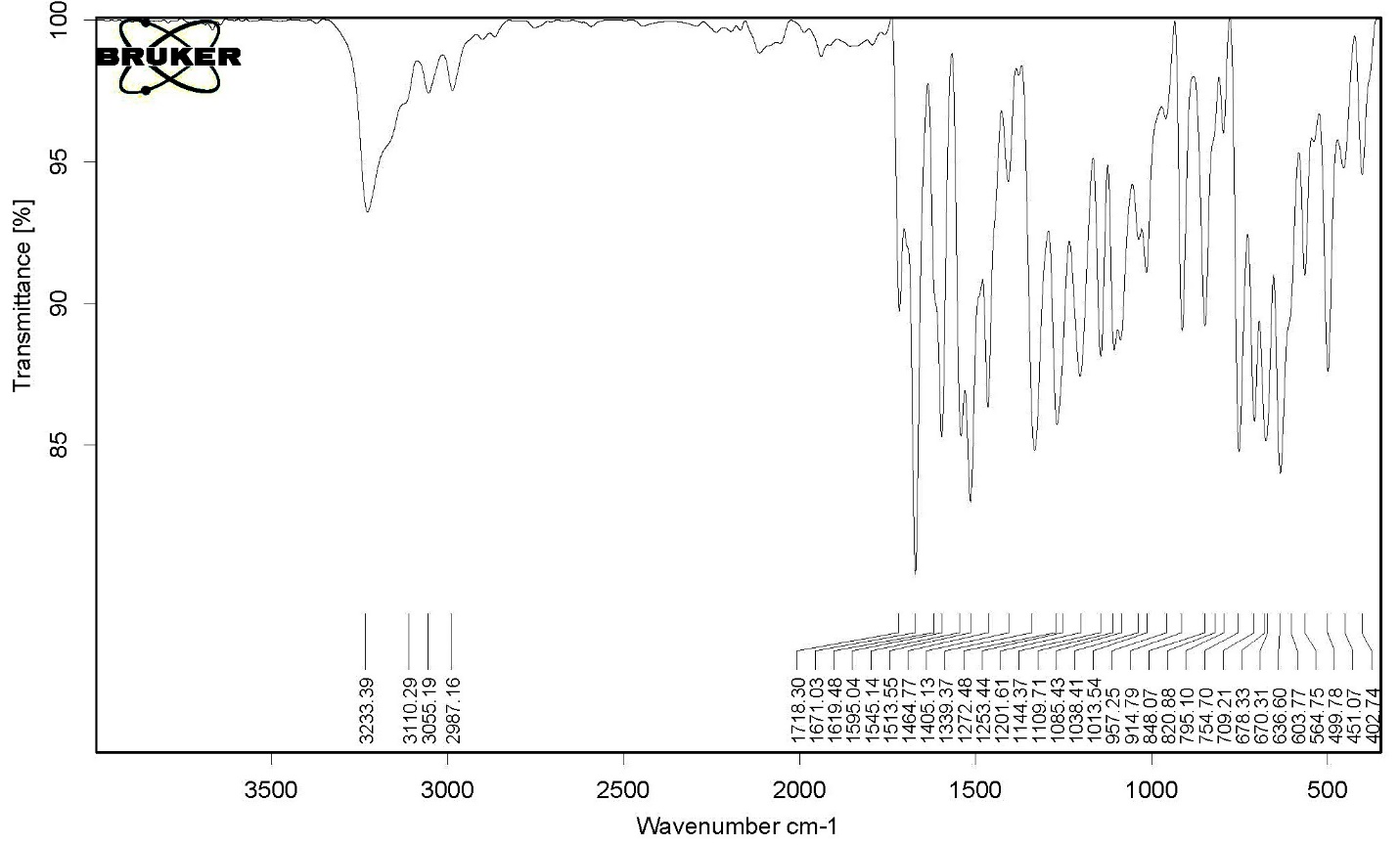
 **Fig (S9): IR spectrum of compound MI-3.**


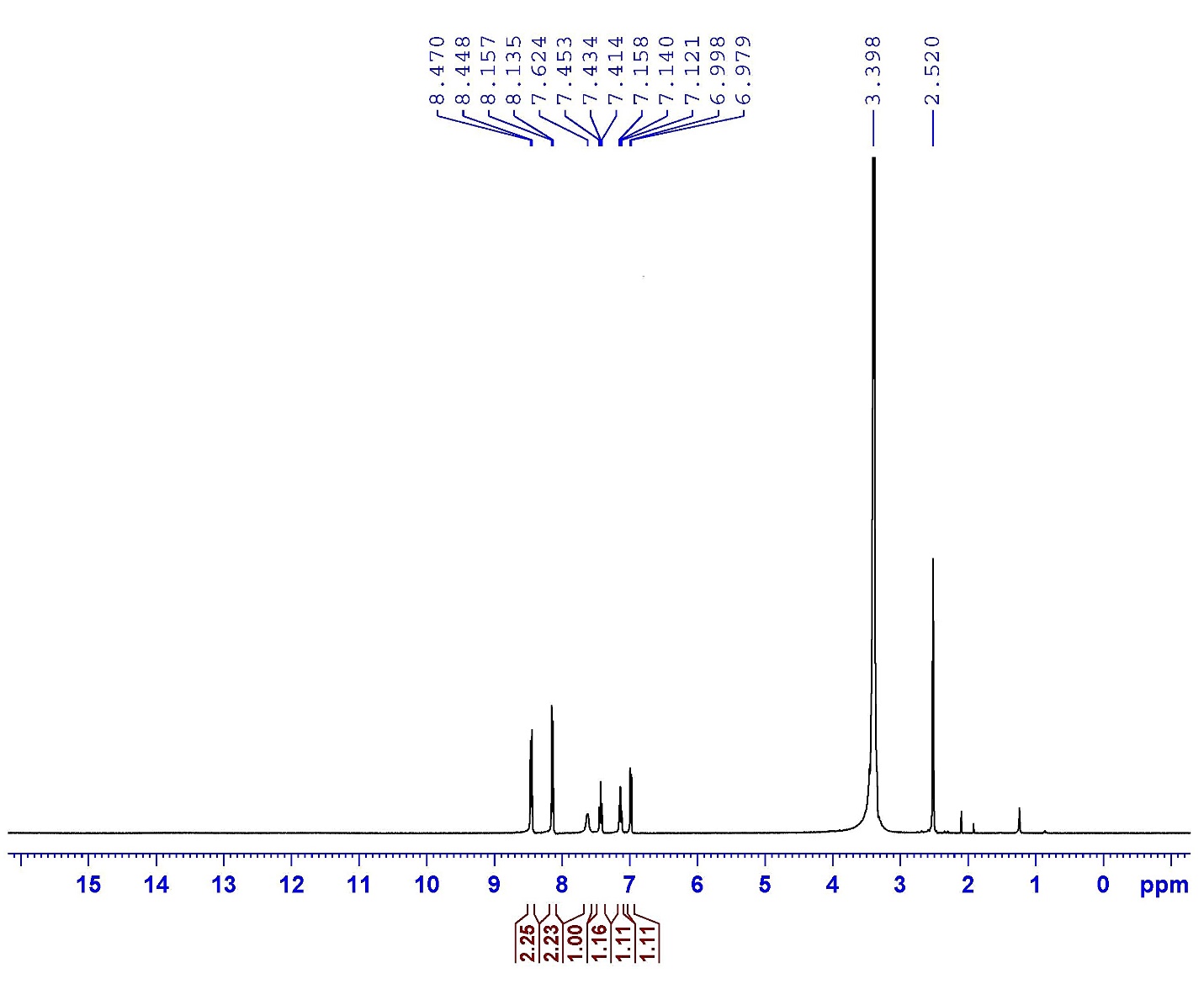


**Fig (S10): ^1^H NMR spectrum of compound MI-3.**


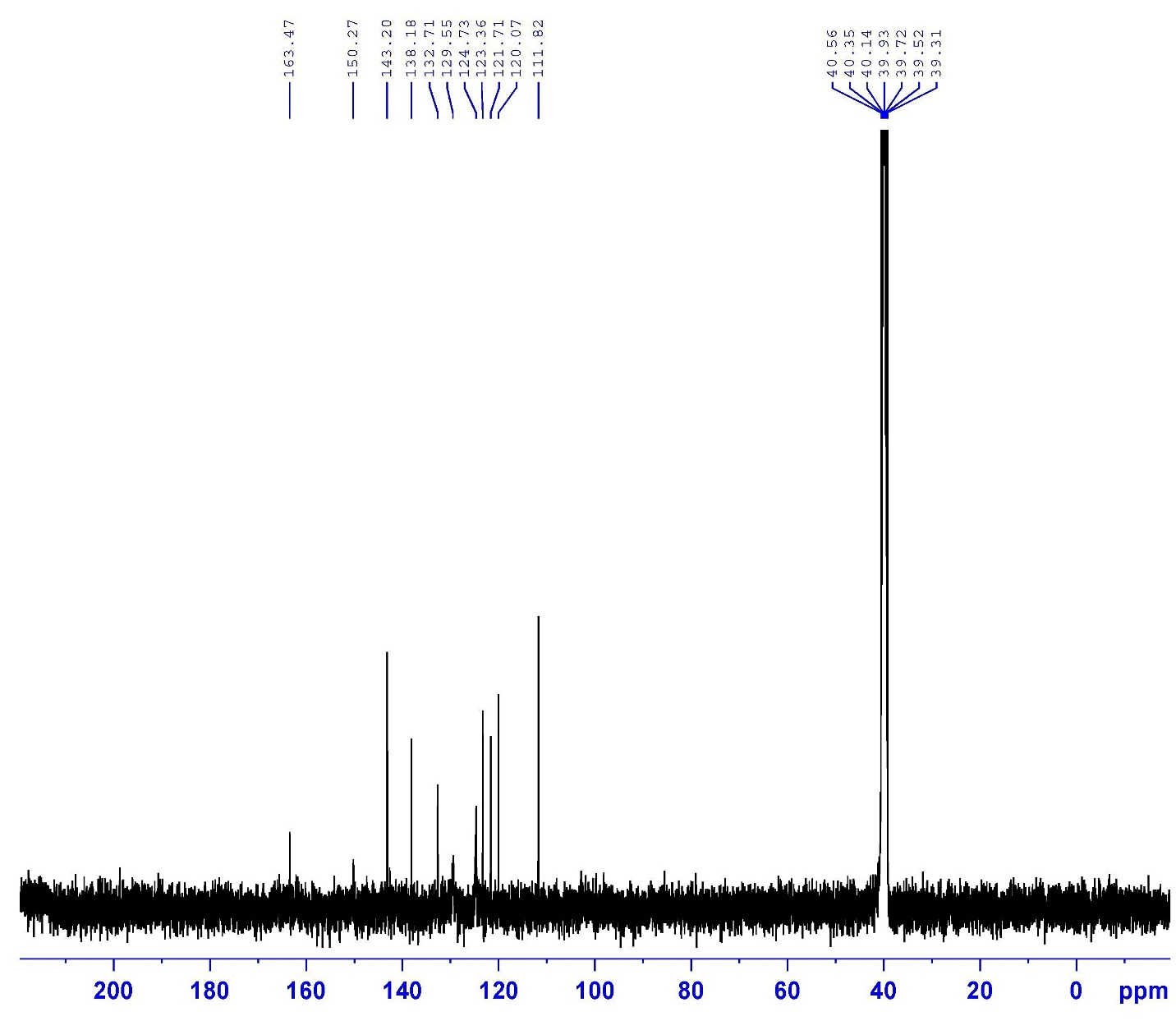
 **Fig (S11): ^13^C NMR spectrum of compound MI-3.**

** Fig (S12): Mass spectrum of compound MI-3.**


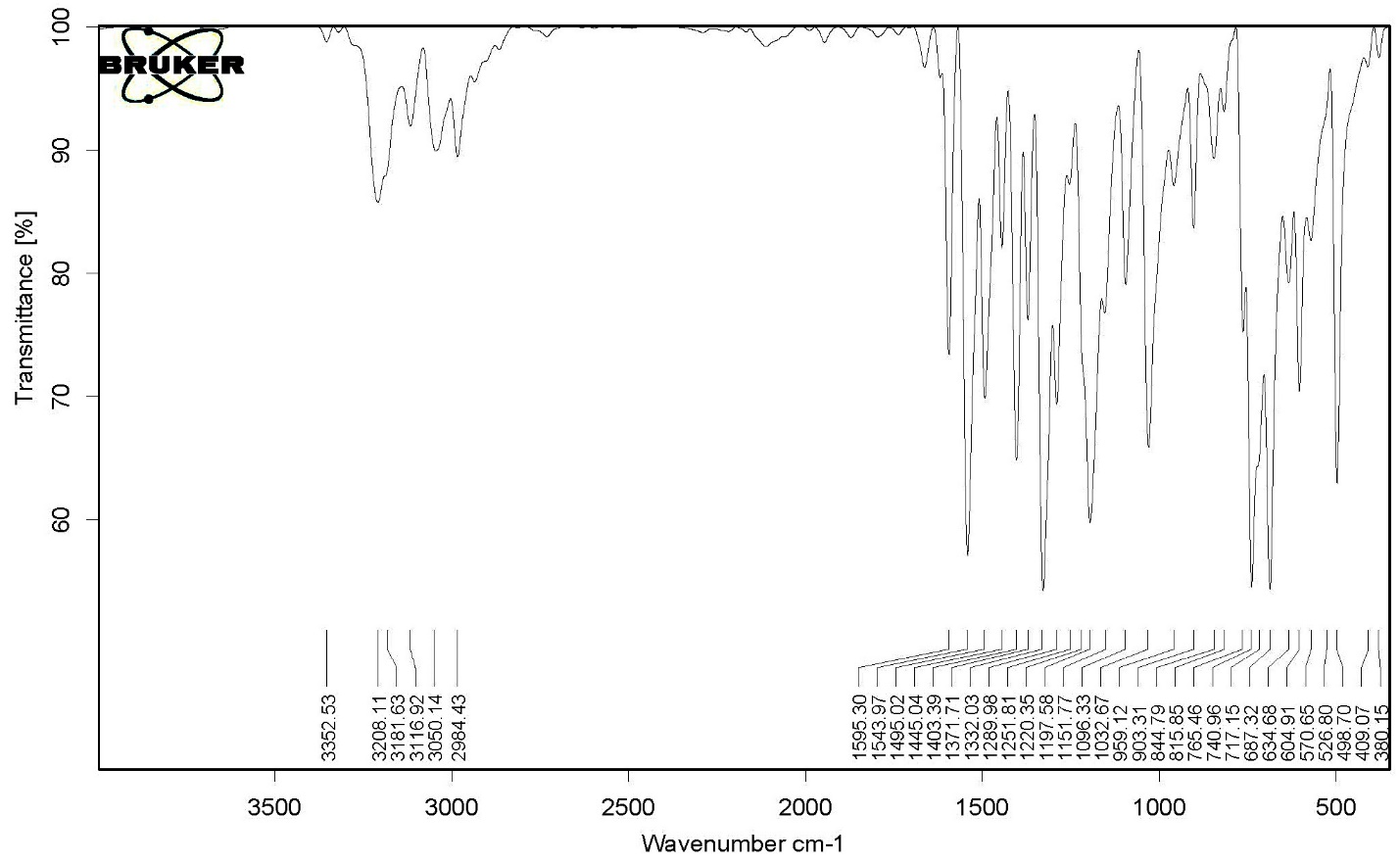
 **Fig (S13): IR spectrum of compound MI-4.**


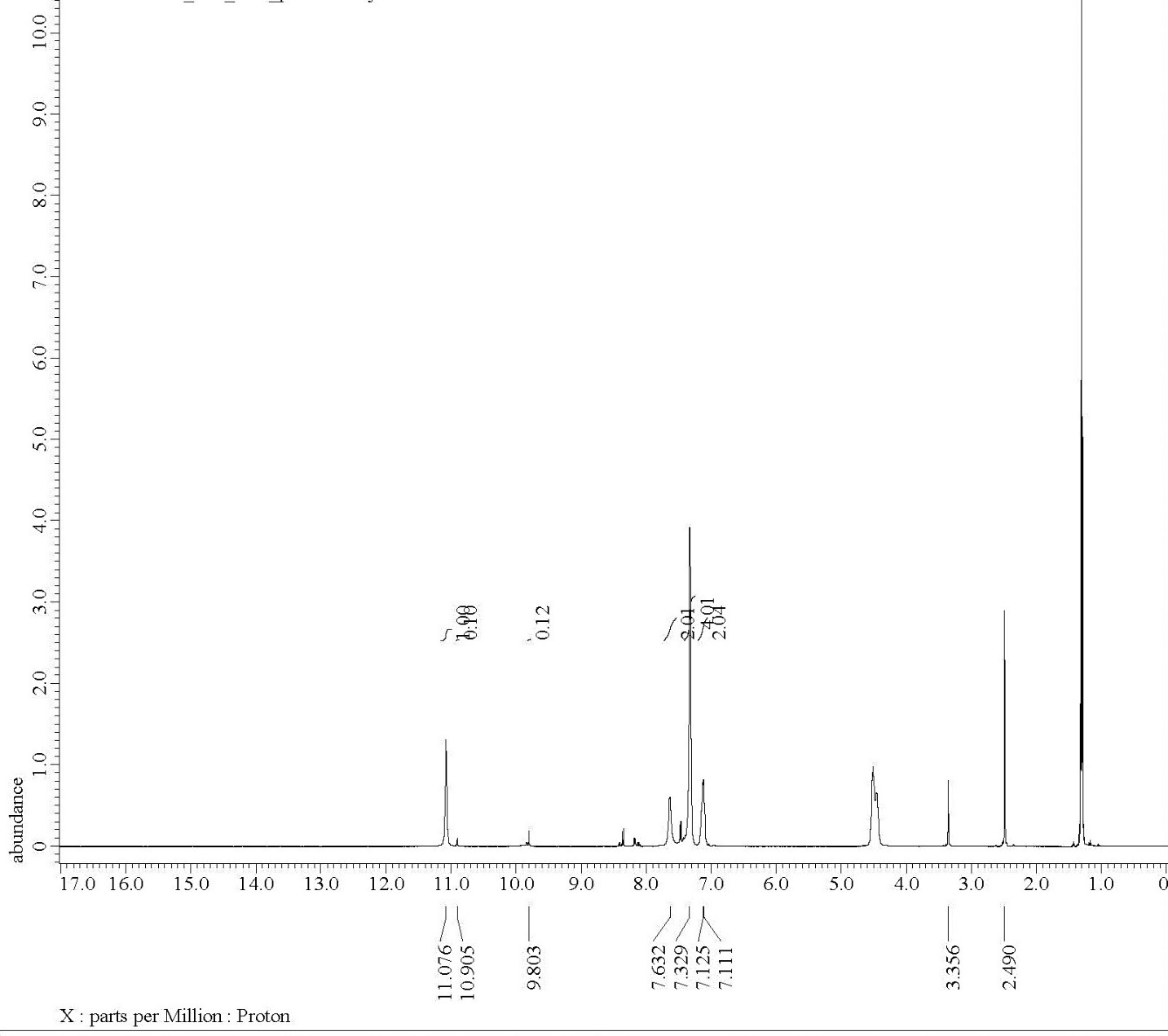


**Fig (S14): ^1^H NMR spectrum of compound MI-4.**

**Fig (S15): Mass spectrum of compound MI-4.**


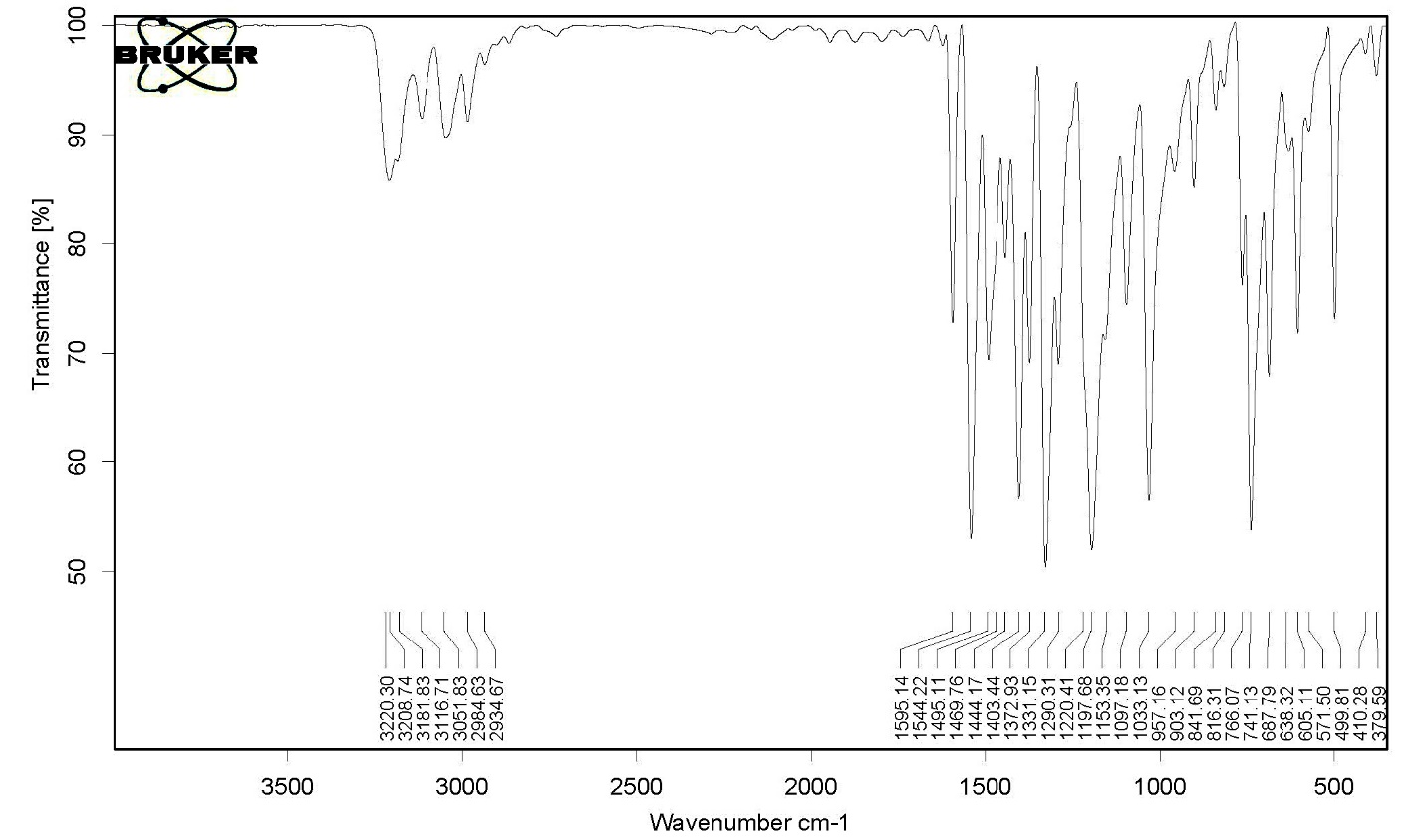
 **Fig (S16): IR spectrum of compound MI-5.**


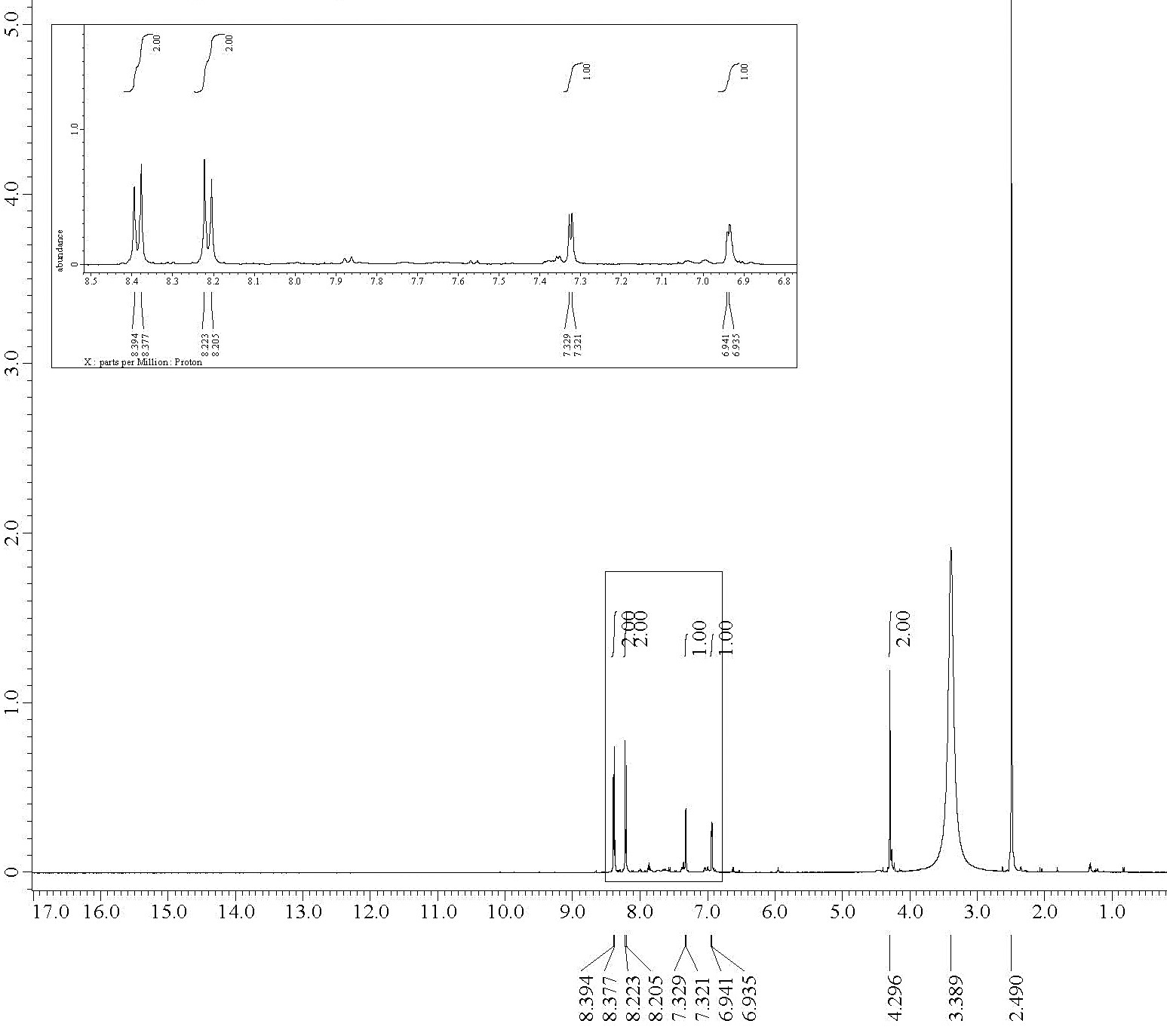
 **Fig (S17): ^1^H NMR spectrum of compound MI-5.**


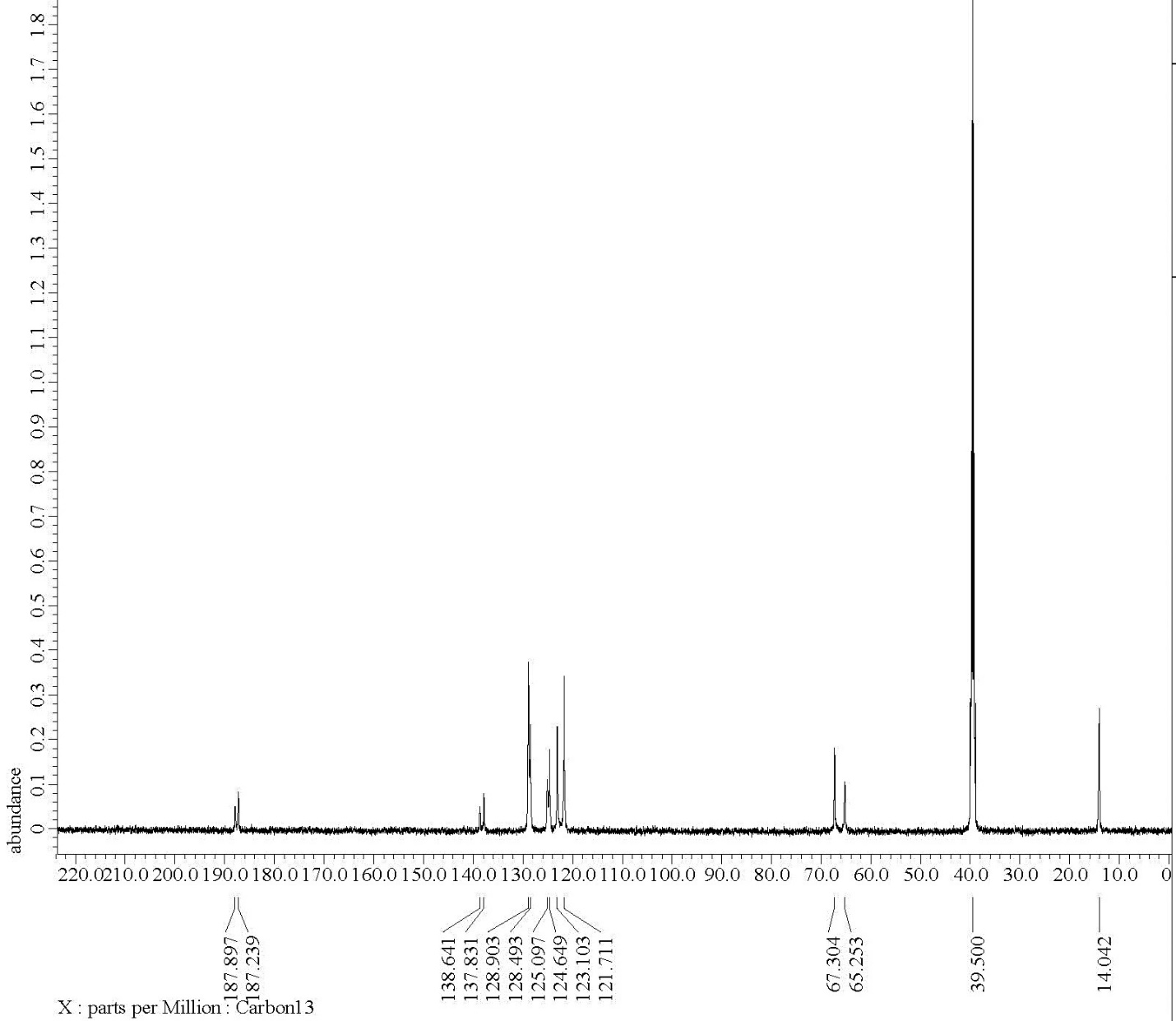


**Fig (S18): ^13^C NMR spectrum of compound MI-5.**

 **Fig (S19): Mass spectrum of compound MI-5.**


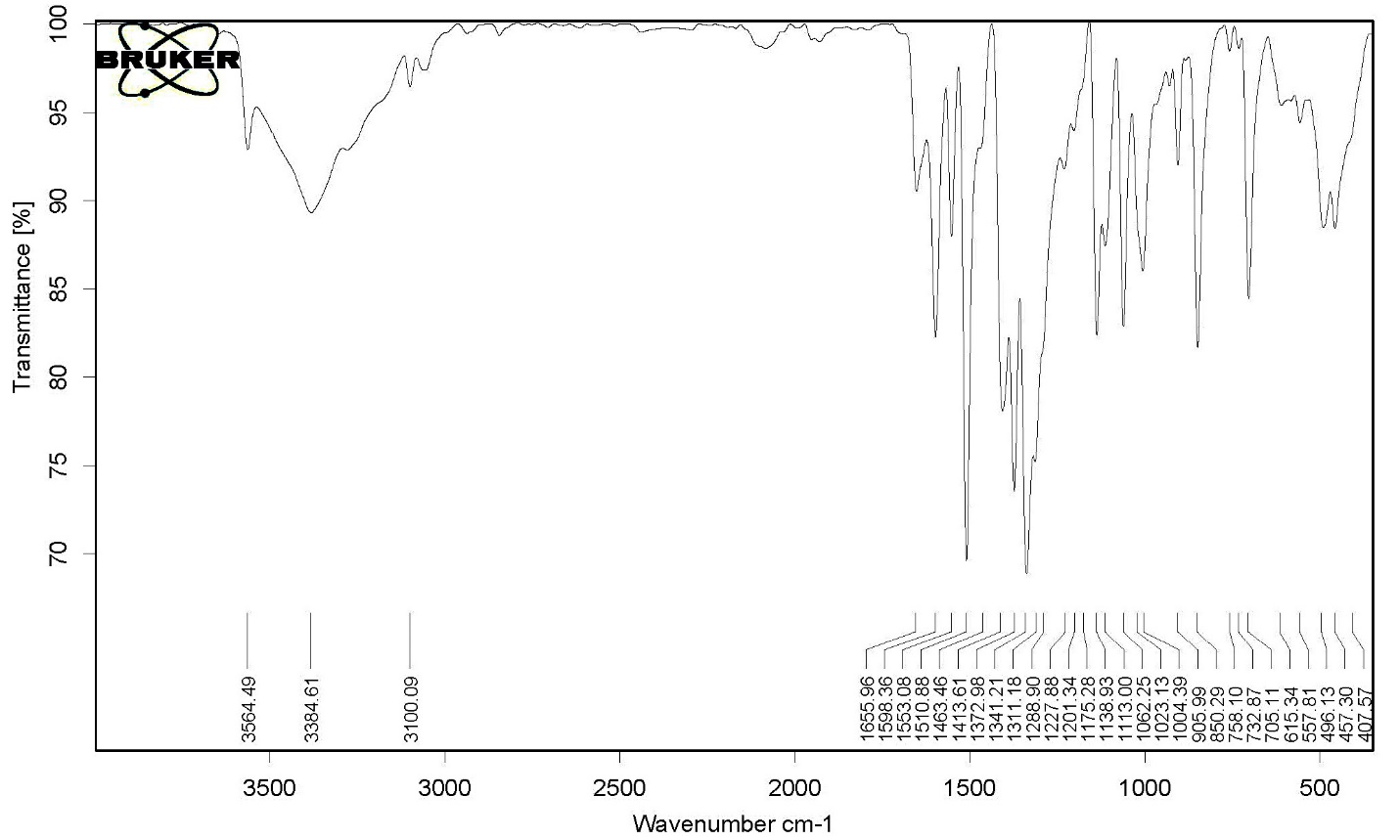
 **Fig (S20): IR spectrum of compound MI-6.**


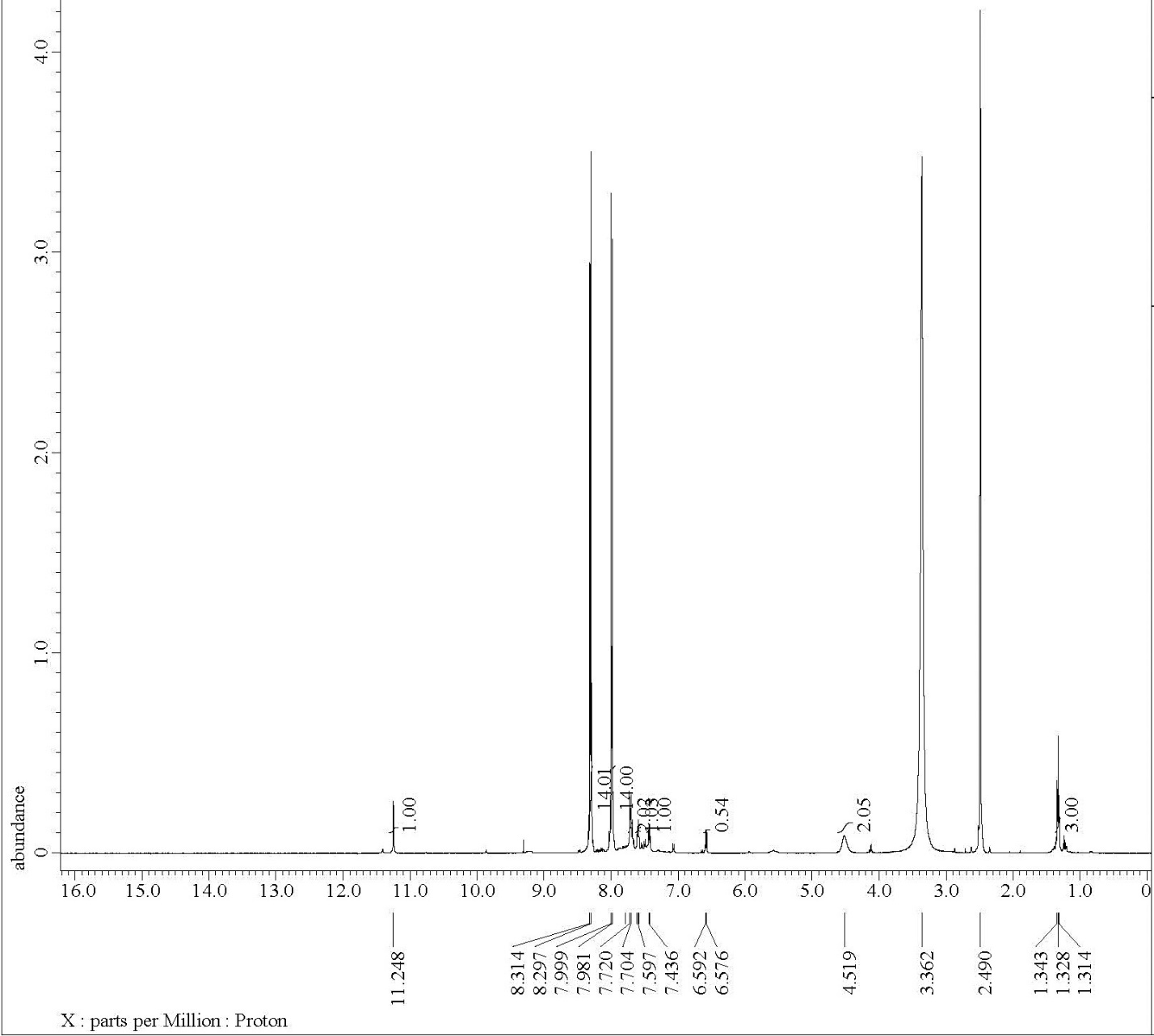
 **Fig (S21): ^1^H NMR spectrum of compound MI-6.**


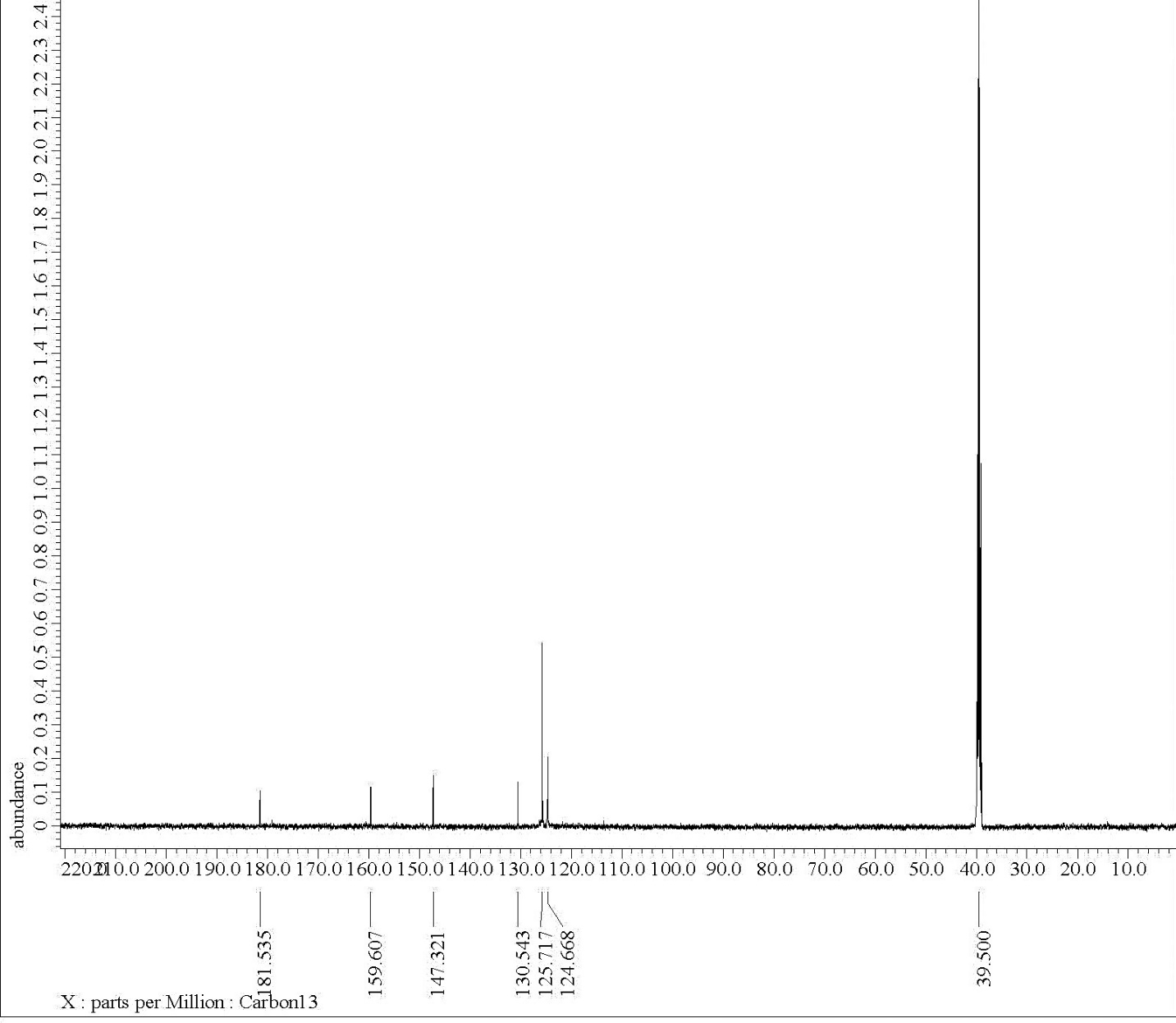
 **Fig (S22): ^13^C NMR spectrum of compound MI-6.**

 **Fig (S23): Mass spectrum of compound MI-6.**


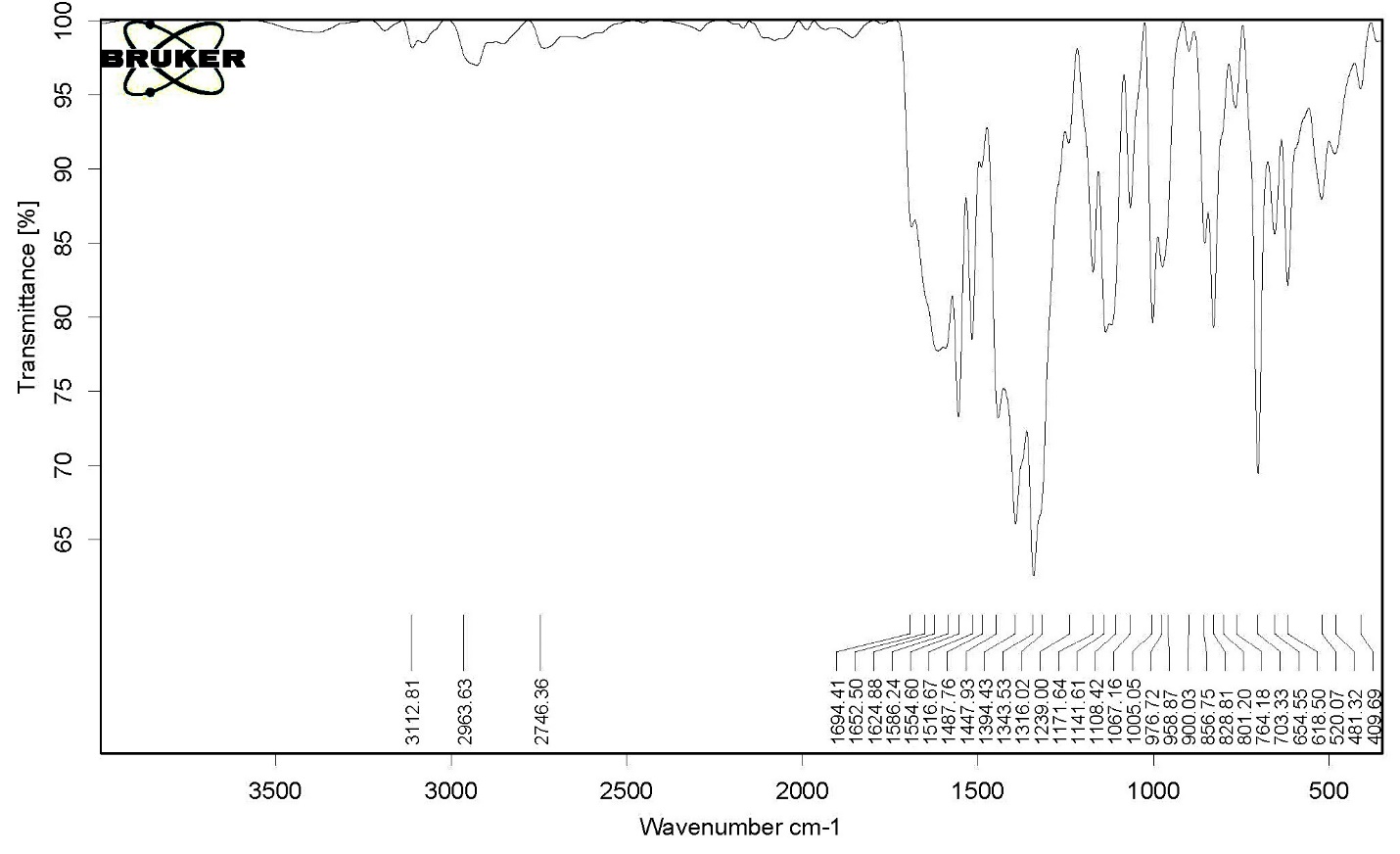


**Fig (S24): IR spectrum of compound MI-7.**


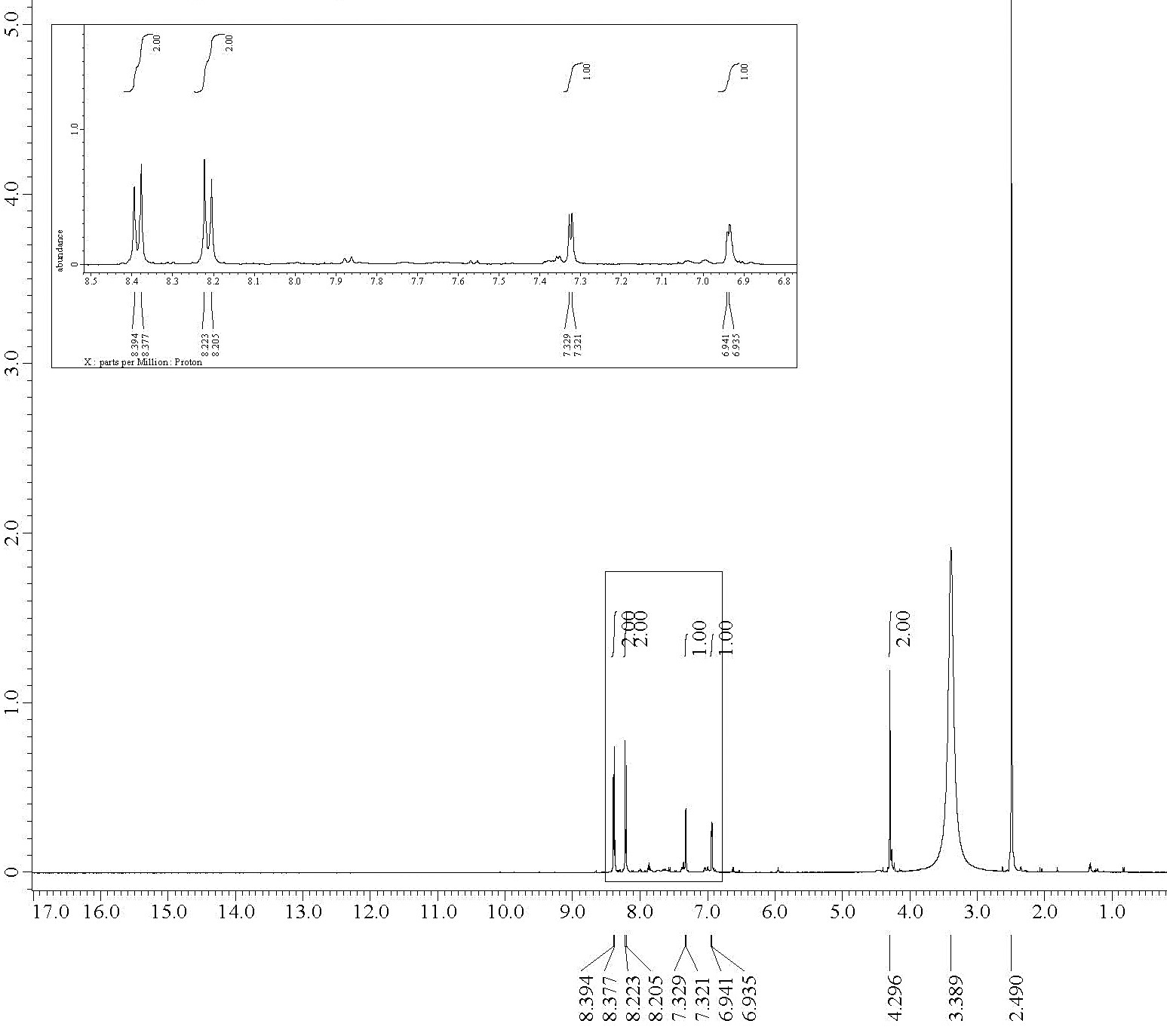
 **Fig (S25): ^1^H NMR spectrum of compound MI-7.**


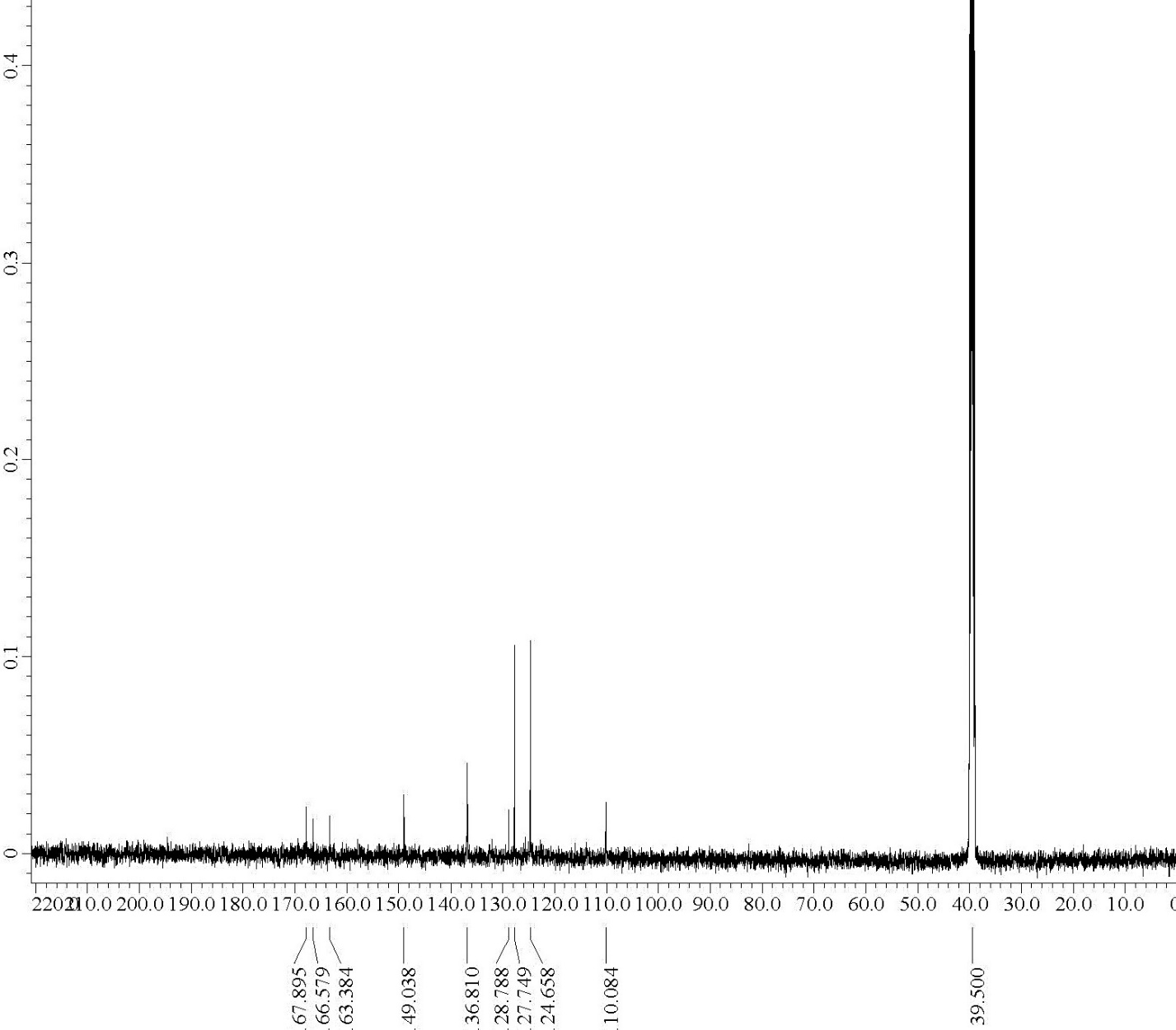
 **Fig (S26): ^13^C NMR spectrum of compound MI-7.**

**Fig (S27): Mass spectrum of compound MI-7.**


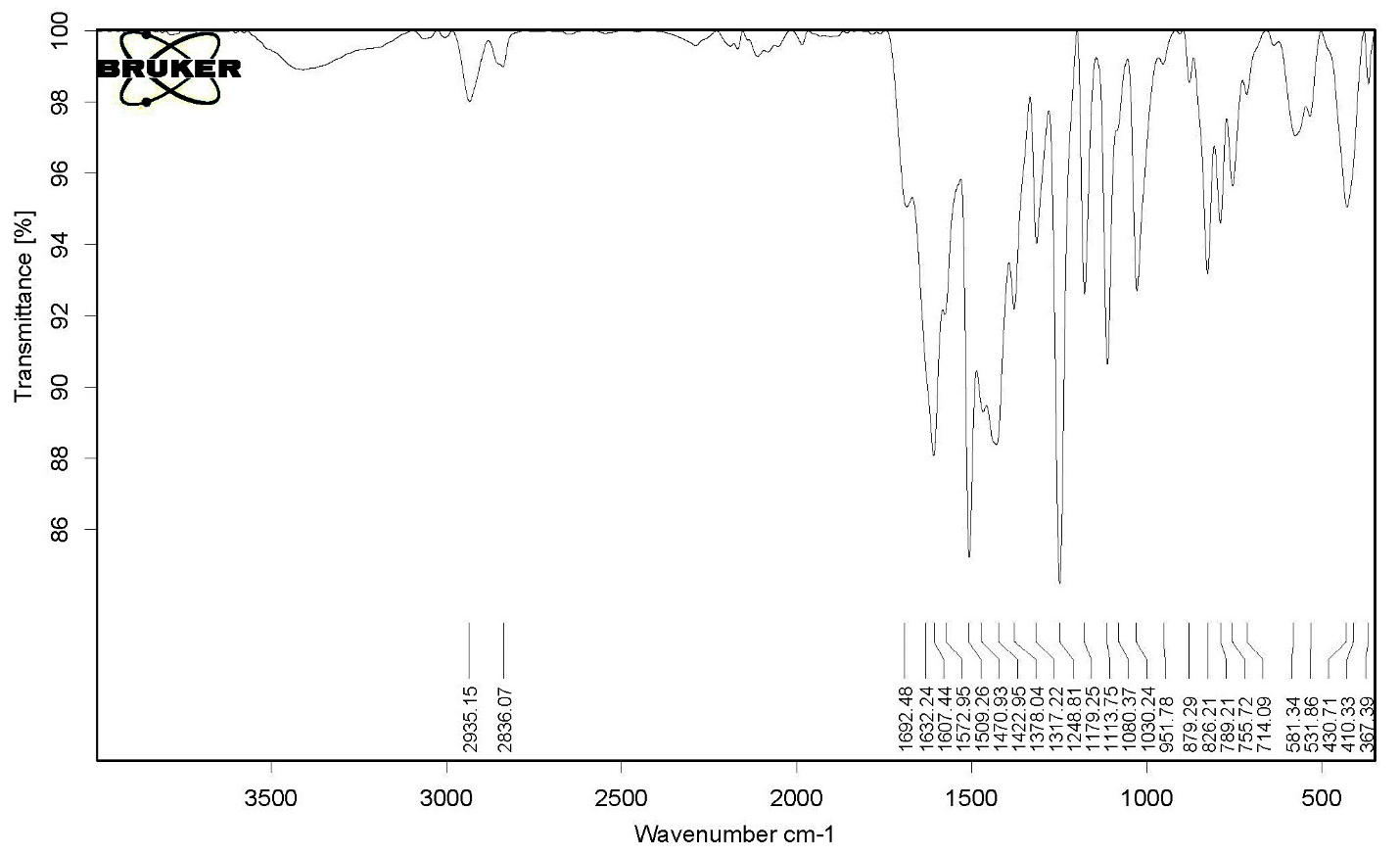
 **Fig (S28): IR spectrum of compound MI-8.**


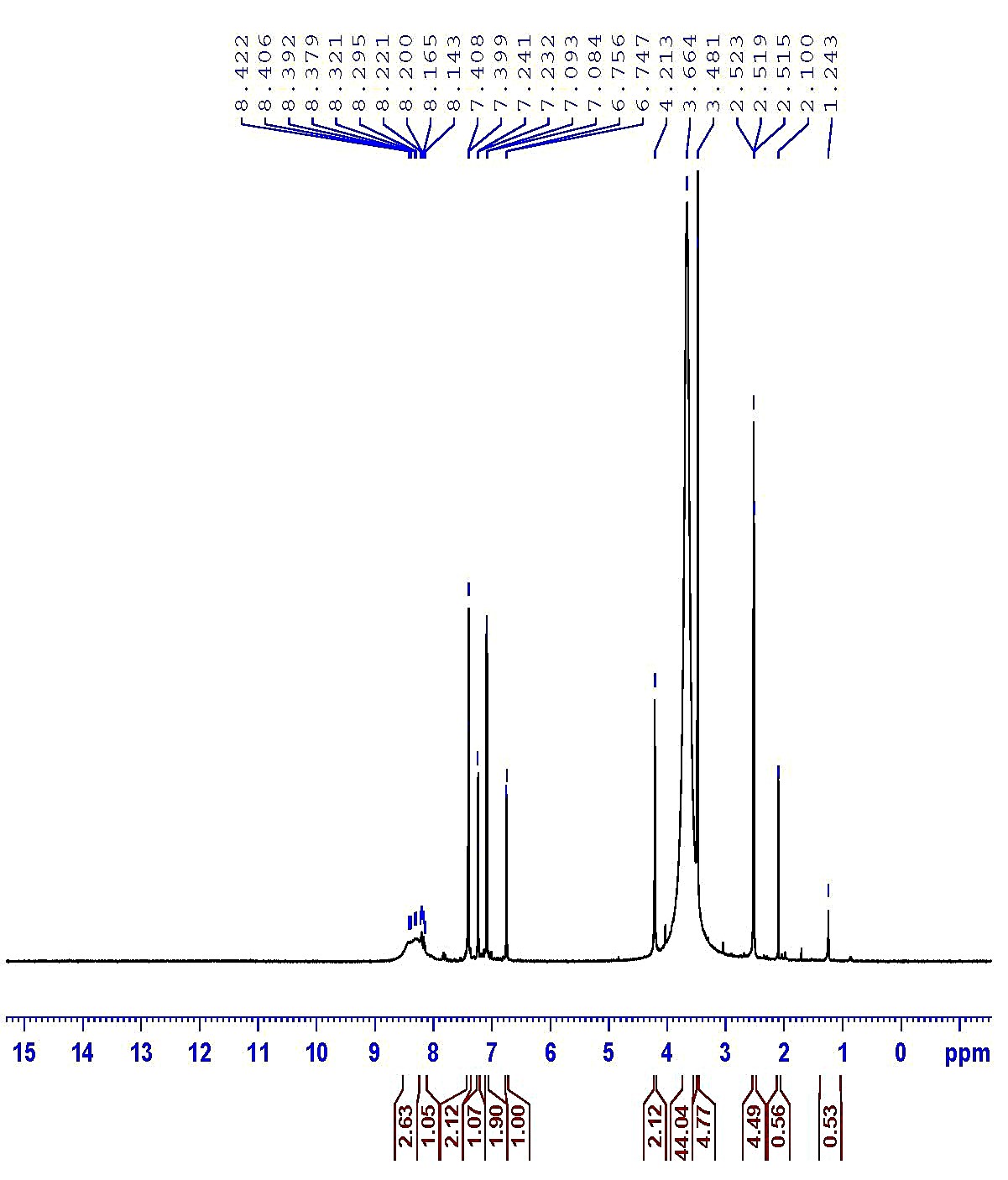
 **Fig (S29): ^1^H NMR spectrum of compound MI-8.**


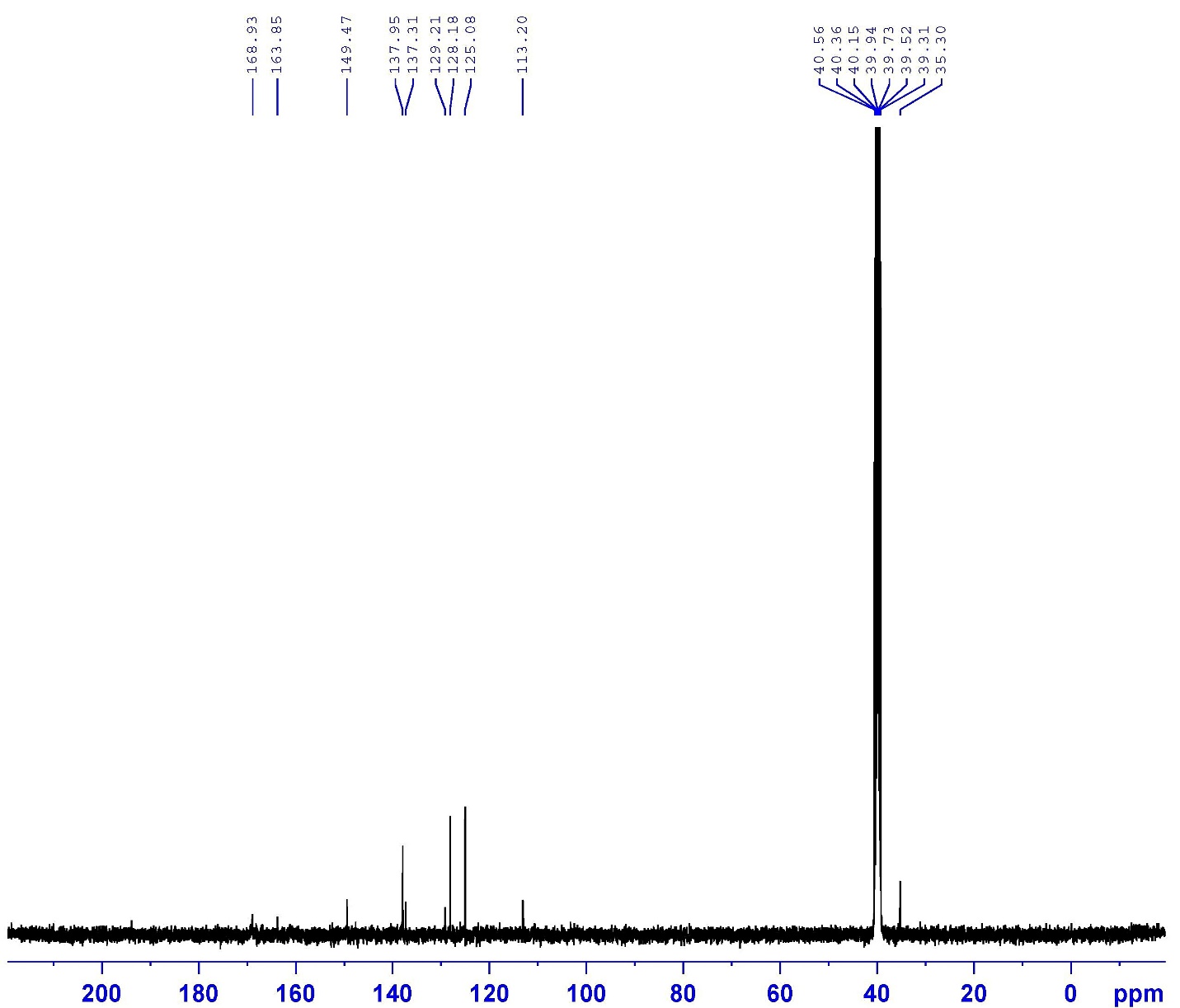


**Fig (S30): ^13^C NMR spectrum of compound MI-8.**

** Fig (S31): Mass spectrum of compound MI-8.**


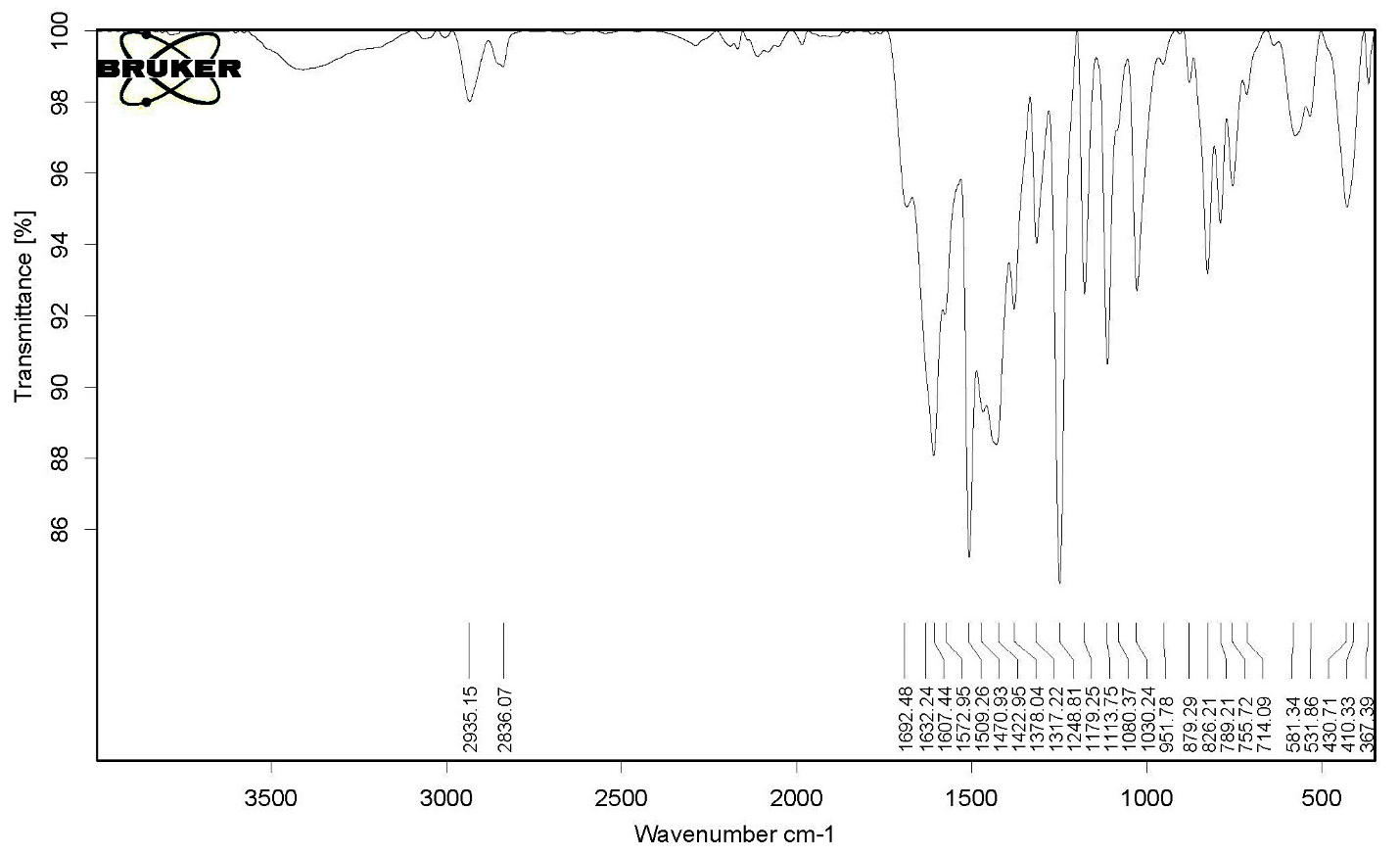
 **Fig (S32): IR spectrum of compound MI-9.**


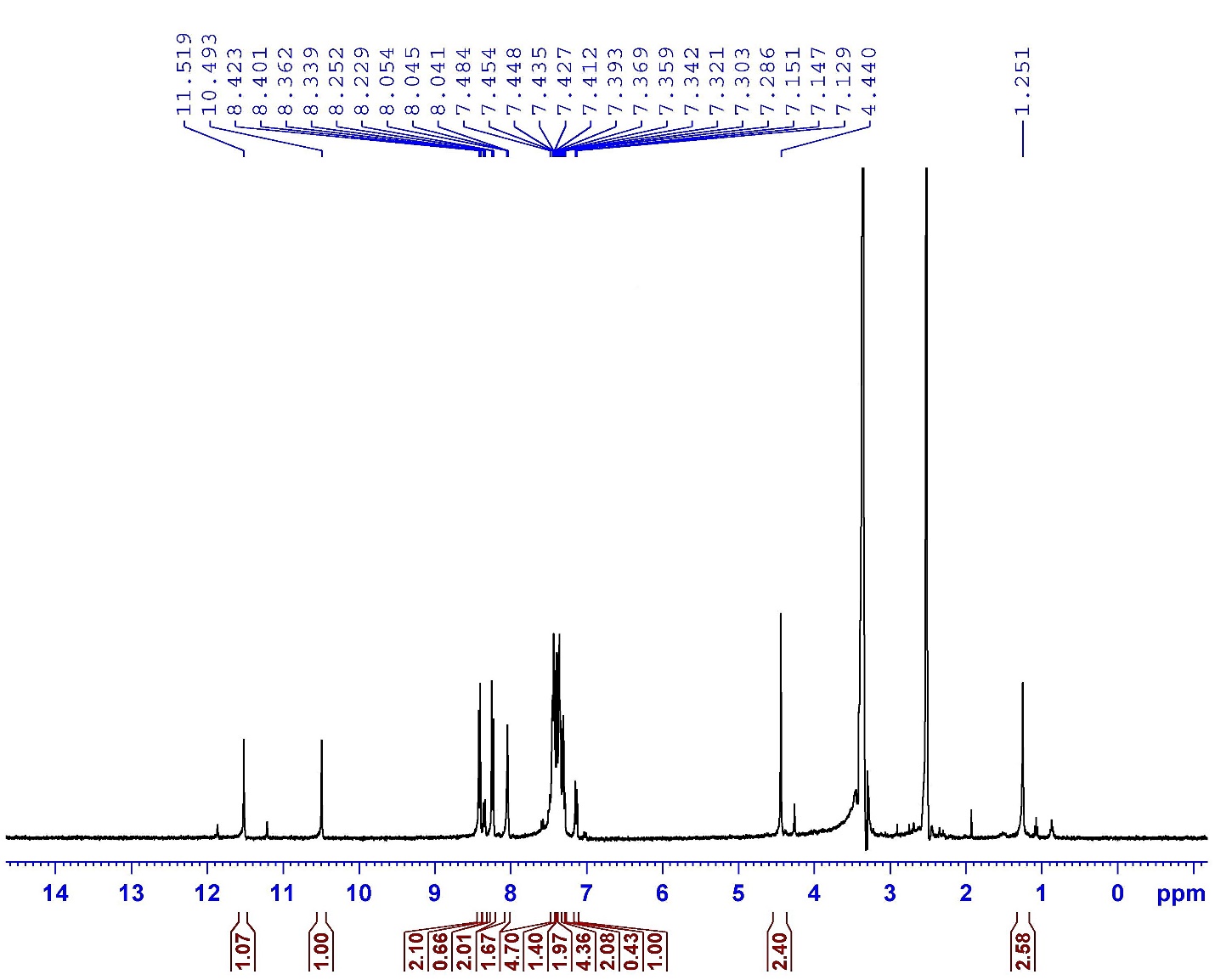
 **Fig (S33): ^1^H NMR spectrum of compound MI-9**


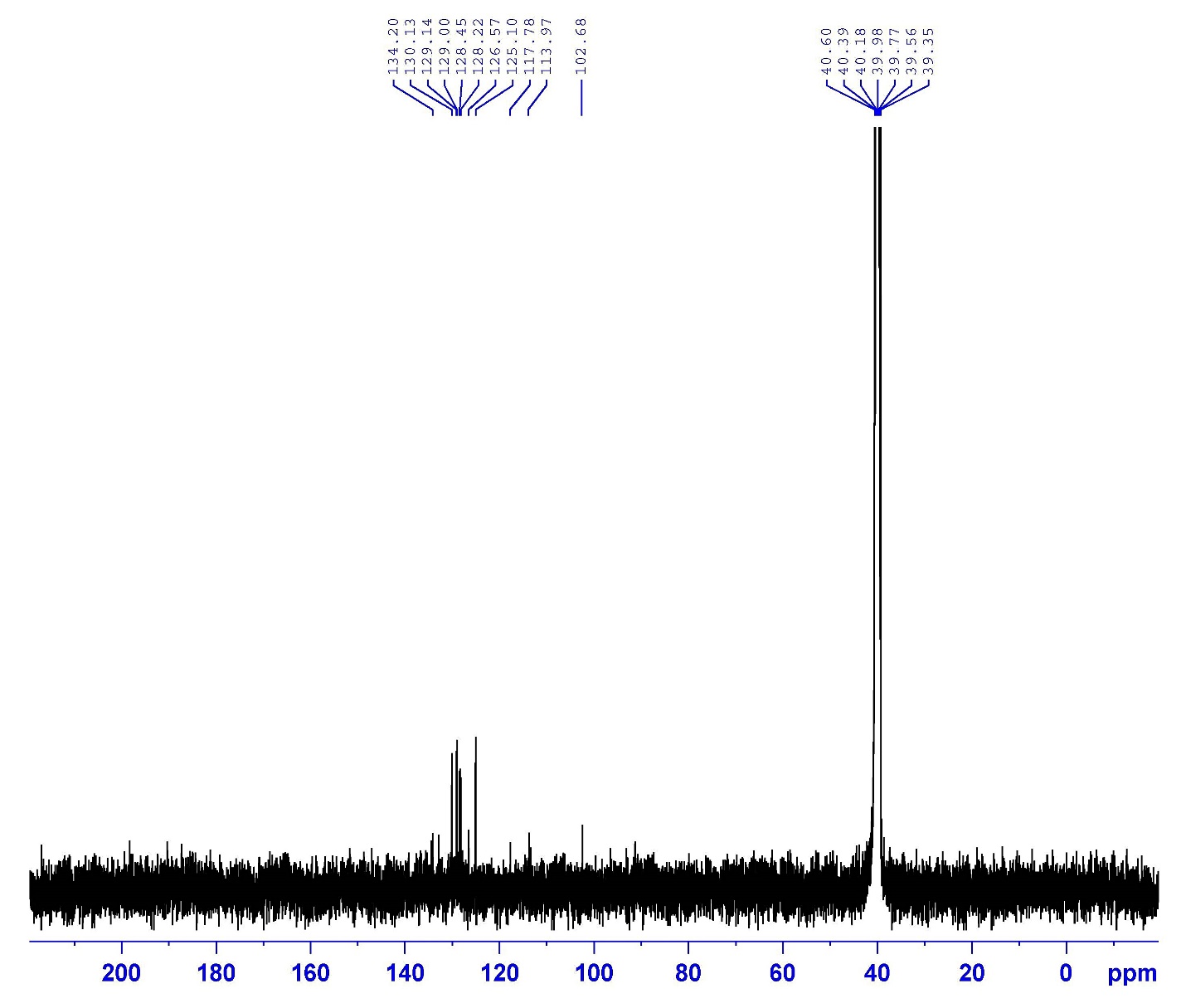


**Fig (S34): ^13^C NMR spectrum of compound MI-9.**

** Fig (S35): Mass spectrum of compound MI-9.**
